# Mitogenome analyses elucidate the evolutionary relationships of a probable Eocene wet tropics relic in the xerophile lizard genus *Acanthodactylus*

**DOI:** 10.1038/s41598-021-83422-7

**Published:** 2021-03-01

**Authors:** Sebastian Kirchhof, Mariana L. Lyra, Ariel Rodríguez, Ivan Ineich, Johannes Müller, Mark-Oliver Rödel, Jean-François Trape, Miguel Vences, Stéphane Boissinot

**Affiliations:** 1 New York University Abu Dhabi, Abu Dhabi, Saadiyat Island United Arab Emirates; 2 Instituto de Biociências, Departamento de Biodiversidade and Centro de Aquicultura (CAUNESP), Universidade Estadual Paulista, Rio Claro, SP CEP 13506–900 Brazil; 3 Institute of Zoology, University of Veterinary Medicine of Hannover, Bünteweg 17, 30559 Hannover, Germany; 4 Institut de Systématique, Évolution, Biodiversité (ISYEB), Muséum national d’Histoire naturelle, CNRS, Sorbonne Université, École Pratique des Hautes Études, Université des Antilles, CP 30, 57 rue Cuvier, 75005 Paris, France; 5 Museum für Naturkunde, Leibniz Institute for Evolution and Biodiversity Science, Invalidenstr. 43, 10115 Berlin, Germany; 6 Laboratoire de Paludologie et Zoologie médicale, IRD, UMR MIVEGEC, B. P. 1386, Dakar, Senegal; 7 Zoological Institute, Technische Universität Braunschweig, Mendelssohnstr. 4, 38106 Braunschweig, Germany

**Keywords:** Ecological modelling, Phylogenetics, Taxonomy, Climate-change impacts, Ecology, Evolution, Ecology, Ecological modelling

## Abstract

Climate has a large impact on diversity and evolution of the world’s biota. The Eocene–Oligocene transition from tropical climate to cooler, drier environments was accompanied by global species turnover. A large number of Old World lacertid lizard lineages have diversified after the Eocene–Oligocene boundary. One of the most speciose reptile genera in the arid Palearctic, *Acanthodactylus*, contains two sub-Saharan species with unresolved phylogenetic relationship and unknown climatic preferences. We here aim to understand how and when adaptation to arid conditions occurred in *Acanthodactylus* and when tropical habitats where entered*.* Using whole mitogenomes from fresh and archival DNA and published sequences we recovered a well-supported *Acanthodactylus* phylogeny and underpinned the timing of diversification with environmental niche analyses of the sub-Saharan species *A. guineensis* and *A. boueti* in comparison to all arid *Acanthodactylus*. We found that *A. guineensis* represents an old lineage that splits from a basal node in the Western clade, and *A. boueti* is a derived lineage and probably not its sister. Their long branches characterize them—and especially *A. guineensis*—as lineages that may have persisted for a long time without further diversification or have undergone multiple extinctions. Environmental niche models verified the occurrence of *A. guineensis* and *A. boueti* in hot humid environments different from the other 42 arid *Acanthodactylus* species. While *A. guineensis* probably remained in tropical habitat from periods prior to the Eocene–Oligocene boundary, *A. boueti* entered tropical environments independently at a later period. Our results provide an important baseline for studying adaptation and the transition from humid to arid environments in Lacertidae.

## Introduction

The world’s biota is largely influenced by the environment, including climate and its change over time and space (e.g.^1^). Across the tree of life, diversity patterns of organisms are linked to climate, although the details of these patterns differ among major groups^2–5^. Present and past climatic conditions are particularly influential in shaping diversity, distribution, physiology and molecular evolution of ectothermic animals, as recently exemplified in lizards of the family Lacertidae^6^.

This family of Old World lizards is the most diverse and ubiquitous squamate group in the western Palearctic, and in addition colonized almost the whole of Asia and the African mainland^6–9^. The recent resolution of the timing of species diversification of the Lacertidae opened the door for more rigorous analyses of evolution, biogeography and species-environment-relationships in this model group^6^, yet a number of species relationships within Lacertidae remain unresolved and await clarification.

Within Lacertidae, the tribe Eremiadini occurs in Africa and arid southwest and central Asia, and the estimated crown age of this clade at 57.1 MYA coincides with one of the warmest period of the Cenozoic^6, 10^. Around that time, the most basal lineage that contains the extant relic taxon of the tribe, *Atlantolacerta andreanskyi*, split from all other Eremiadini^11–13^. The remaining Eremiadini diverged into two major clades roughly corresponding to the geographic distribution of their respective members, the Saharo-Eurasian clade and the Ethiopian clade^7, 9^. The Eocene climatic optimum at 52-50 MYA was followed by a 17-MY-long trend toward cooler and drier conditions, which was accompanied by global species turnover and resulted in what is called the Eocene–Oligocene extinction event between the end of the tropical Eocene at ~ 33.7 MYA and the beginning of the cooler and drier Oligocene^10^. A large number of lacertid lineages have adapted to the cooler temperatures after the Eocene–Oligocene boundary^6^.

Among these lineages, the genus *Acanthodactylus* Wiegmann, 1834 is one of the most diverse and widespread diurnal reptiles in the arid regions from the Iberian Peninsula through North Africa, Arabia and the Middle East to western India. It is the most species-rich genus in the family Lacertidae with currently 44 recognized species inhabiting a wide variety of dry habitats^14, 15^. Most of its extant species diversity originated in the Miocene^6, 14^. The genus is generally referred to as arid-adapted and sand-living^14, 16–18^, exceptionally also occupying more compact soils^14, 18^ and even relatively mesic habitats (i.e. the *A. erythrurus* group with *A. erythrurus* (Schinz, 1833), *A. savignyi* (Audouin, 1827), *A. boueti* Chabanaud, 1917 and *A. guineensis* (Boulenger, 1887) (fide^18^).

Within the Saharo-Eurasian clade of Eremiadini^7, 9^ *Acanthodactylus* belongs to a subclade containing three other mainly arid Palearctic genera (*Eremias*, *Mesalina*, *Ophisops*), with *Ophisops* also reaching the Oriental zoogeographic region. Herein, we propose calling this subclade the ‘Northern arid clade’. The Northern arid clade is sister to what has been named the ‘Equatorial African clade’ comprising the genera *Adolfus*, *Congolacerta*, *Gastropholis*, *Holaspis*^7^, herein renamed to ‘Equatorial African-Arabian clade’ as it also includes the two relic species of *Omanosaura* from the Arabian Peninsula^6^. From the Northern arid clade, only two *Acanthodactylus* taxa—*A. boueti* and *A. guineensis*—occur exclusively south of the Sahara. These represent a biogeographic enigma, especially because their phylogenetic relationships remain largely unresolved^6, 14, 19^ despite enormous progress in *Acanthodactylus* systematics largely triggered by recent molecular analyses^6, 14, 19–26^. While the affiliation of *A. boueti* to the Western clade of *Acanthodactylus* is relatively well established despite an unstable phylogenetic position^6, 14^, the relationship of *A. guineensis* remains uncertain. Different trees placed the species either at unsupported nodes basal to the entire Northern arid clade containing *Acanthodactylus*, *Eremias*, *Mesalina* and *Ophisops*^6^, basal to the *Acanthodactylus* genus (in a BI dated tree^14^) or basal to the *Acanthodactylus* Western clade (in a BEAST consensus tree^14^). This uncertainty is mainly the result of very limited sampling with only 791 base pairs from two mitochondrial gene fragments (12S ribosomal RNA (12S) and cytochrome b (COB)) from a single individual (two individuals in Fonseca et al.^19^, not available on GenBank). Due to the high genetic distance of *A. boueti* and *A. guineensis*, however, it was suggested that the two species are not closely related^14^. This in turn would indicate that either (i) these two species independently entered sub-Saharan and potentially wetter environments after the ancestor of the genus already adapted to desert conditions, or (ii) *A. guineensis,* and maybe also *A. boueti,* are the sister to all other *Acanthodactylus* and the genus thus may have originated in tropical Africa before it adapted to drier conditions and diversified, or (iii) these species, especially the enigmatic *A. guineensis*, do not belong to the genus *Acanthodactylus* at all.

In this study we aimed to understand how and when adaptation to arid conditions occurred in the genus *Acanthodactylus*. In order to do so we first required an extended genetic sampling of one of the hitherto missing key species, *A. guineensis.* Since no fresh material has been collected recently (mainly due to safety and logistic issues affecting the currently politically unstable regions of western and central Africa where the species occurs) we used tissue samples collected from museum specimens that often yield low quality and highly fragmented DNA. Then we applied a shotgun next generation sequencing strategy, a method that is commonly used to obtain sufficient genetic material from low-quality samples for subsequent assembly of whole mitogenomes^27, 28^. We aimed to achieve a reliable understanding of the evolutionary relationships of *Acanthodactylus* spp. in general and resolve the phylogenetic position of *A. guineensis* specifically using whole mitogenomes of *A. guineensis*, *A. schmidti* (for which we had genomic data available from an ongoing study), twelve lacertid taxa assembled over the course of a previous study^6^ and published data on mitogenomes and common mitochondrial marker genes of crucial additional taxa. We updated the distribution range of *A. guineensis* and assembled data on habitat and environmental conditions prevailing within its distribution range. With the improved phylogeny as a backbone, we aimed to compare the environmental niche of both sub-Saharan species *A. guineensis* and *A. boueti* with all arid-adapted *Acanthodactylus* spp. in order to evaluate the climatic history and adaptation of this diverse and widespread genus. We focused especially on *A. guineensis* and hypothesized that it represents an early-derived members of *Acanthodactylus* or even its own genus and has persisted for a long time in tropical environmental conditions.

## Results

### Molecular data and phylogenetic analysis

The nearly complete newly assembled mitogenomes of *A. guineensis* and *A. schmidti* as well as the twelve additional taxa of Gallotiinae and Eremiadini each consist of 13 protein-coding genes, 22 transfer RNA (tRNA) genes, 2 ribosomal RNA (rRNA) genes typical for vertebrates. Gene order was the same as in other known lacertid mitogenomes^29–31^. We also recovered partial sequences of the control region for all mitogenomes. The assembly lengths varied between 15,552 and 17,143 bp (Supplementary Table S1 online). The final mitogenome assemblies of three species presented low coverage regions within genes that were coded as consecutive unidentified bases (N) in the final MITObim output: these included 48 and 29 bp in the COB gene for *A. guineensis* and *A. schmidti*, respectively, and 149 bp comprising the 3′ end of cytochrome c oxidase subunit I gene and the tRNA Serine 2 gene for *A. erythrurus.*

**Figure 1:**
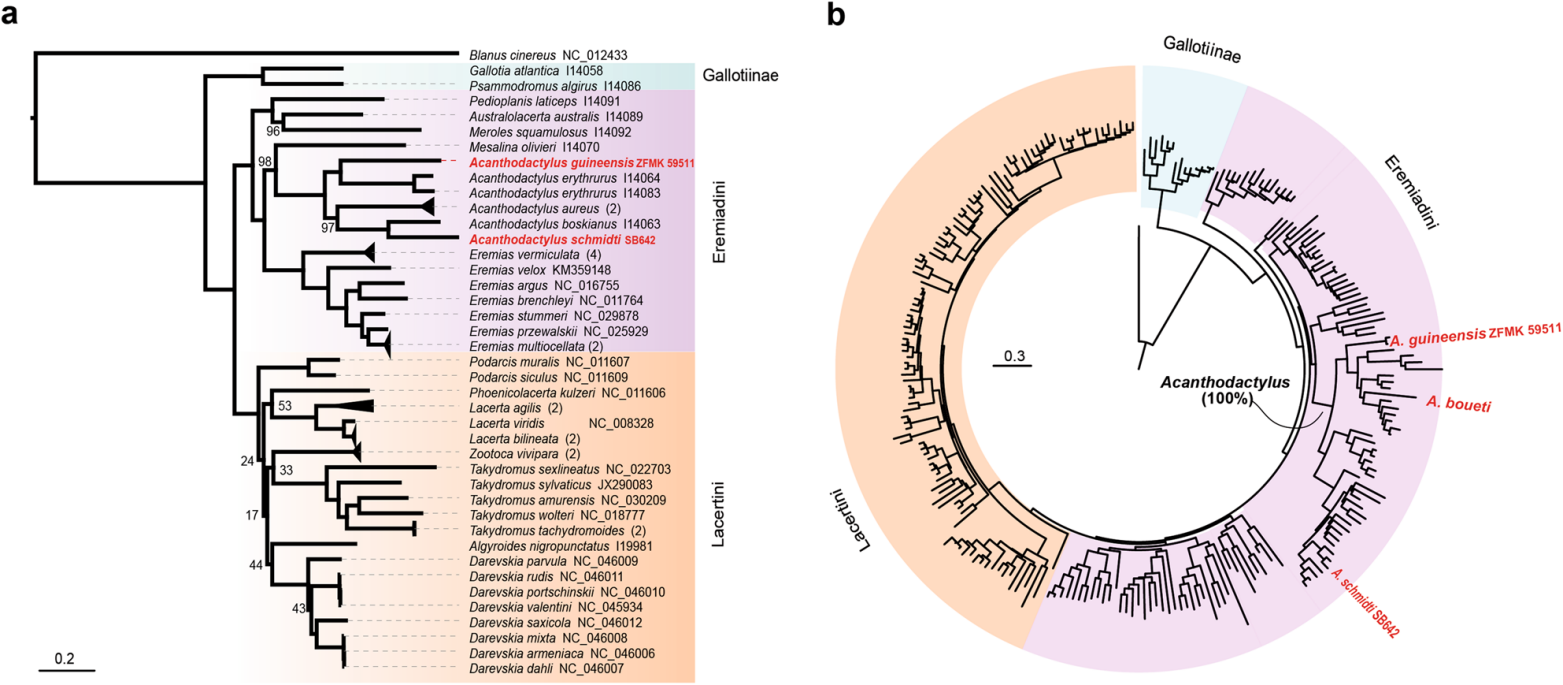
Phylogenetic position of the newly assembled mitogenomes of *Acanthodactylus guineensis* and *A. schmidti*. (**a**) Phylogenetic reconstruction based on a novel mitogenomic alignment of DNA sequences from 50 terminals, including 14,241 sites of 12S and 16S rRNAs plus the 13 protein-coding genes. Numbers in parentheses mark species for which more than one mitochondrial genome was available (see Supplementary Fig. S1 online; collapsed a posteriori for graphical representation), focal taxa are highlighted in red. Branch thickness is proportional to ultrafast bootstrap node support values (only values < 100 are shown). (**b**) Phylogenetic reconstruction based on a more comprehensive alignment, with reduced gene coverage, including 3054 sites of the 12S, 16S, COB and ND4 genes from 250 terminals. Focal taxa are highlighted in red. See Supplementary Material online for additional details on the phylogenies and main text for details of the phylogenetic methods used in both analyses.

For the mitogenomic phylogeny, two rRNA and 13 protein coding genes were used. Both maximum likelihood and Bayesian analyses recovered monophyletic groups corresponding to the two lacertid subfamilies Gallotiinae and Lacertinae, the two Lacertinae tribes Lacertini and Eremiadini, as well as the two main Eremiadini clades, the Saharo-Eurasian clade (represented here by the genera *Mesalina*, *Acanthodactylus* and *Eremias*) and the Ethiopian clade (here represented by *Meroles*, *Pedioplanis*, *Australolacerta*) with high support^6, 7, 9, 32, 33^; Fig. 1a, Supplementary Figs. S1, S2 online). The only difference between the two trees were the positions of the Lacertini genera *Podarcis* and *Algyroides* which switched places (with moderate node support). Within the Eremiadini the majority of nodes received 100% ultrafast bootstrap node support values (posterior probabilities = 1) and no node was supported with less than 96%. The tree further confirms specimen ZFMK 59511 from Burkina Faso to be a member of the genus *Acanthodactylus* (i.e., *A. guineensis* based on the morphological identification, see Supplementary Material online) with 100% node support (Fig. 1a, Supplementary Fig. S1 online). Specifically, *A. guineensis* was recovered sister to a clade containing the two members of the *erythrurus* group included in the phylogenetic tree, and these three species together were sister to a clade containing *A. aureus, A. boskianus* and the *A. schmidti* specimen from Abu Dhabi (Fig. 1a, Supplementary Figs. S1, S2 online).

**Figure 2:**
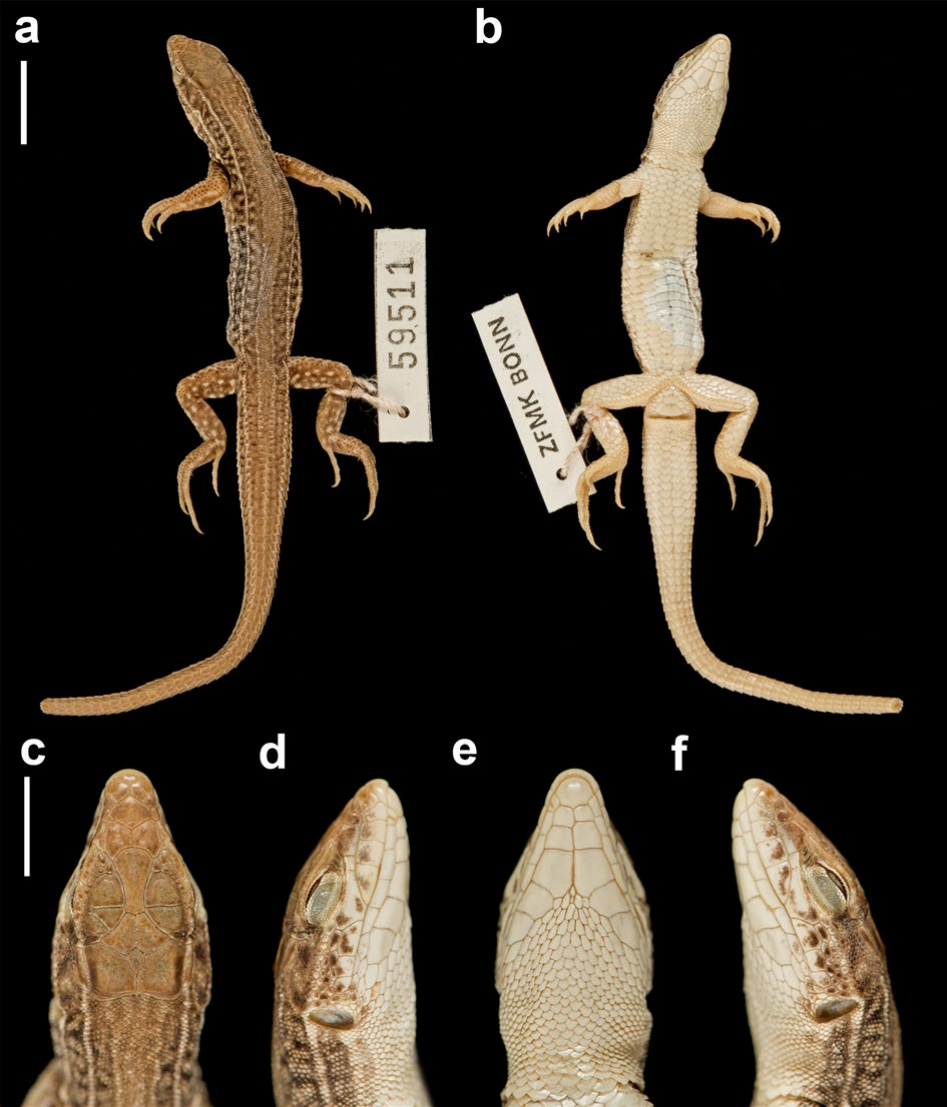
Photographs of the newly sequenced specimen of *A. guineensis* (ZFMK 59511) with dorsal and ventral whole animal shots and head close ups (dorsal, ventral and both laterals). The white line represents 1 cm (top) and 0.5 cm (bottom), respectively. Photographs taken by M. Flecks (ZFMK).

The maximum likelihood analyses based on more comprehensive taxon sampling with 250 terminals but using only 3054 sites from the most common mitochondrial markers 12S, 16S, COB, ND4 (Fig. 1b, Supplementary Fig. S3 online), as expected, was unable to correctly recover deep phylogenetic relationships within lacertids (for such relationships, we here rely on the phylogenomic reconstructions of Garcia-Porta et al.^6^). However, the tree is informative at more shallow nodes, and it reconstructed with high support the previously established relationships of most *Acanthodactylus* species included. Specifically, the three major clades Western, Eastern and *scutellatus*^6, 14, 26^ were recovered and confirmed with high node support (99–100%; Fig. 1b, Supplementary Fig. S3 online). Our main target taxon, *Acanthodactylus guineensis* was placed with full bootstrap support into the Western clade, where it was sister to all other species, i.e., the North African *A. savignyi* (sometimes considered member of the *erythrurus* group^14^)*,* the Middle Eastern *tristrami* group (*A. robustus, A. orientalis, A. tristrami*)*,* and the African *erythrurus* group (*A. blanci, A. erythrurus*) and *pardalis* group (*A. boueti, A. margaritae, A. busacki, A. bedriagai, A. maculatus*)*.* The newly sequenced specimen of *A. guineensis* (ZFMK 59511 from Daroha, near Bobo Dioulasso, Burkina Faso; Fig. 2) for which all gene fragments were available, clustered closely with the previously sequenced specimen (A275 = JFT 4143 from Kouré, Niger) for which only two fragments were sequenced, and which therefore could not be reliably placed into the lacertid tree in previous studies.

The second sub-Saharan *Acanthodactylus*, *A. boueti* was not recovered as sister taxon of *A. guineensis* based on the available sequences of 12S and COB from GenBank (accession number A276) but instead placed with high support (bootstrap value 99) as sister to the *pardalis* group within the Western clade.

The individual of *A. schmidti* (SB 642) from Abu Dhabi (UAE) clustered with *A. schmidti* (KJ652757.1) from Iran^24^ and was sister to *A. blanfordii* in the so-called *blanfordii* group^14^.

### Distribution and habitat

Over the course of this study, we discovered undetected specimens of *Acanthodactylus guineensis* in the collection of ZMB (Supplementary Fig. S4 online). One of them (ZMB 25479) is the rediscovered holotype of *Eremias mandjarum* Sternfeld, 1916, which based on morphological characters we consider to be a synonym of *A. guineensis*^34, 35^ and not of *Heliobolus nitidus*^36^. Morphological data on the new specimens is provided in the Supplementary Material online.

**Figure 3:**
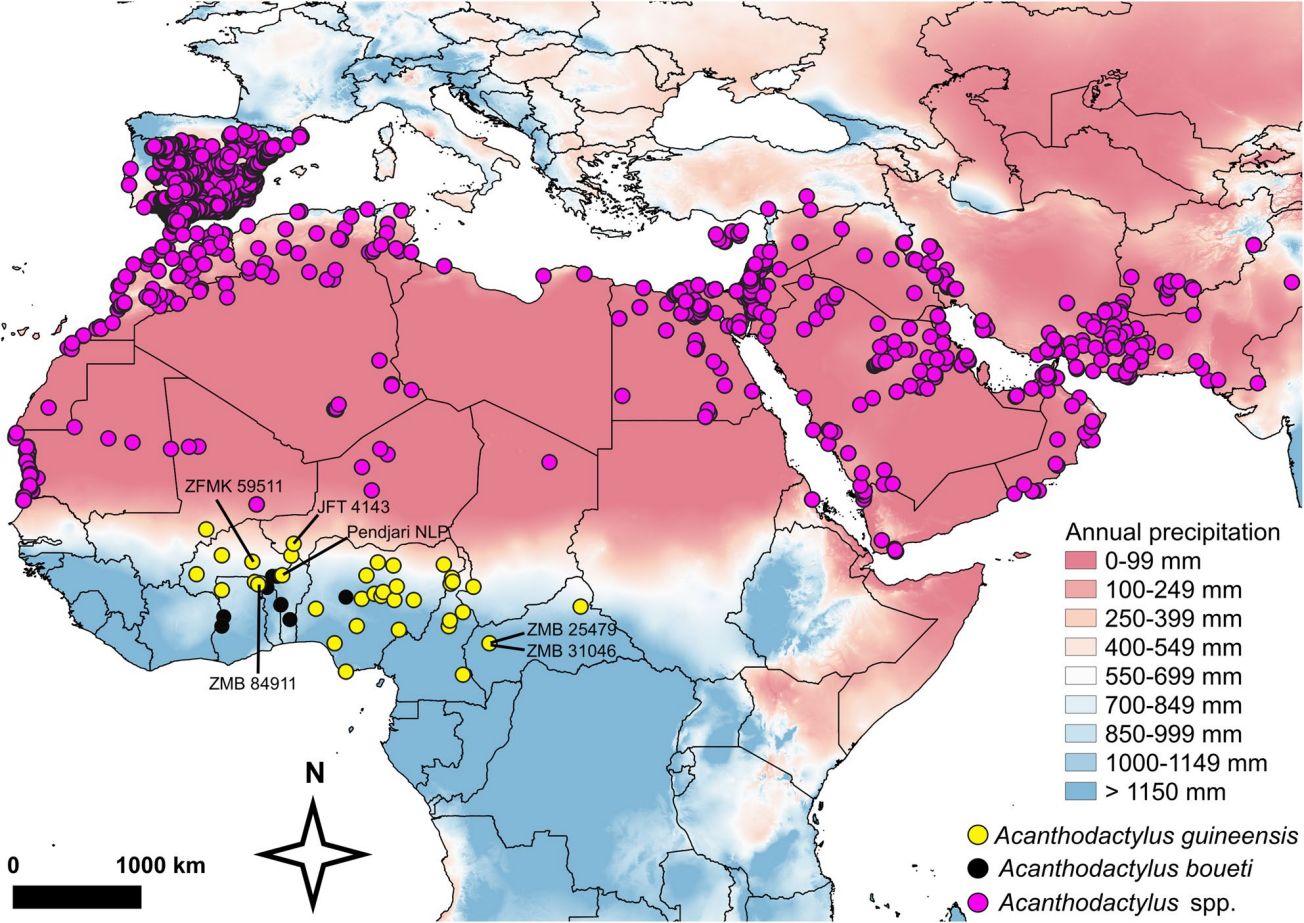
Updated distribution map for *Acanthodactylus guineensis* (yellow circle﻿) and *A. boueti* (black circle) including all literature and GBIF records and our own discoveries from this study (unpublished). In addition, we present all records from the genus *Acanthodactylus* (purple circle) as downloaded from GBIF. The specimen that we sequenced (ZFMK 59511^43^), museum specimens studied herein (holotype *E. mandjarum* ZMB 25479, ZMB 31046, ZMB 84911), the previously unpublished country record for Benin^38^, and the *A. guineensis* specimen collected by JFT (4143, GenBank record A275^14^) that we added to our phylogeny are separately labeled on the map. In the background, data on mean annual precipitation in mm is shown, with cooler colors representing wetter conditions and warmer colors representing drier conditions. The figure was created using QGIS 3.14.16 (https://www.qgis.org).

After including the new records, the updated distribution range of *Acanthodactylus guineensis* comprises the following West and Central African countries: Mali, Burkina Faso, Ghana, Benin (first country record from Pendjari National Park^37, 38^, Chirio pers. comm.), Niger, Nigeria, Cameroon, and Central African Republic (Fig. 3). After reducing the locality dataset to one record per km^2^ to avoid pseudoreplication and testing for clustering we kept 36 separate, randomly distributed localities for the habitat analyses and species distribution model (Supplementary Table S2 online).

**Figure 4:**
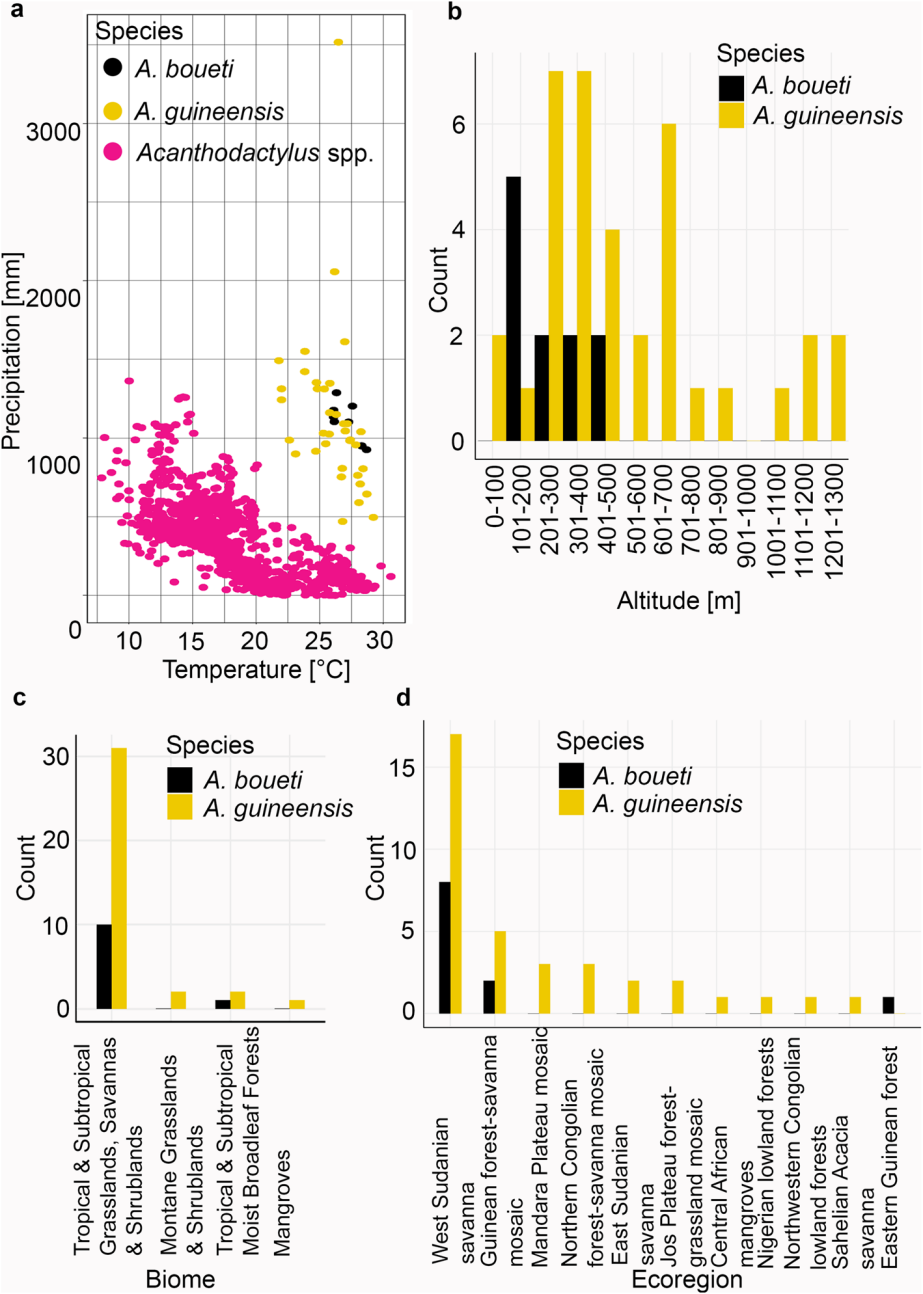
(**a**–**d**) Environmental niche and habitat of *A. guineensis* and *A. boueti* with respect to (**a**) mean annual temperature and precipitation in comparison to all other *Acanthodactylus* spp., (**b**) elevation, (**c**) inhabited biomes, and (**d**) ecoregions.

Compared to the other 42 species of *Acanthodactylus*, *A. guineensis* and *A. boueti* inhabit wet and warm habitats and there is no climatic niche overlap between the two sub-Saharan species and all xerophile species with regards to annual temperatures and precipitation (Fig. 4a).

Of the 36 known *A. guineensis* localities, 58.3% are found in elevations between 0 and 500 m, and another 27.8% between 501 and 900 m; five localities (13.9%) are above 1000 m (range 7–1207 m; Fig. 4b). The median average annual temperature is 26.5 °C (range 21.9–29.1 °C), median average annual precipitation is 1027 mm (range 488–3515 mm).

According to our analysis, *A. guineensis* occurs in four different biomes^39^, most of which fall into the ‘Tropical & Subtropical Grasslands, Savannas and Shrublands’ category (86.1% of occupied habitats), but others are in the ‘Montane Grasslands & Shrublands’ and ‘Tropical & Subtropical Moist Broadleaf Forests’ biomes (Fig. 4c). The one locality from the ‘Mangroves’ biome is the area where the type locality “Brass, mouths of the Niger”^40^ is located, however, various authors have cast doubt on the correctness of the type locality and expect it to be further north^18, 41^. This locality also stands out with experiencing the highest annual precipitation in our dataset (3515 mm; see Fig. 4a), while all other environmental parameters recorded fall within the total range for this species. Almost half of the known populations (47.2%) are found in the ecoregion ‘West Sudanian savanna’, another 27.8% are located in different forests and forest-savanna mosaics (Fig. 4d). Specimen JFT 4143 was collected in typical Sahelian vegetation on sand with scattered acacia trees and a few dry herbaceous plants. A Nigerian individual from the Jos plateau was found on rocky substrate with small trees and dry herbs at 1324 m elevation (JFT pers. obs.); however, this specimen was not caught and while it is known to occur on Jos Plateau^42^ the rocky habitat appears unusual (but see below). Since there is a chance for misidentification when seen from a distance, we decided to exclude this specimen from our habitat analyses. According to the literature, the majority of habitats of *A. guineensis* are sand dominated and located near water (e.g. near rivers Niger, Yamé, or a sandy patch near a fountain or in peanut plantations) but there are occasional records also from harder soil and rocky habitat structures^43–45^.

In comparison, *A. boueti* is known from eleven localities in Ghana, Togo, Benin and Nigeria. Here, it inhabits mainly the ‘Tropical & Subtropical Grasslands, Savannas and Shrublands’ biome (90.9%, ecoregions ‘West Sudanian savanna’ and ‘Guinean forest-savanna mosaic’) while one record is probably from ‘Tropical & Subtropical Moist Broadleaf Forests’ (‘Eastern Guinean forests’). However, the coordinates of the latter locality have low accuracy (see “Methods”) and could potentially also be located in ‘Guinean forest-savanna mosaic’. The species range covers much lower elevations than *A. guineensis* (from 161 to 419 m, median 203 m) where it experiences a slightly warmer median average annual temperature (27.3 °C) and slightly higher median average annual precipitation (1085 mm) (Fig. 4a–d).

### Species distribution models

The data assembled show that in contrast to the majority of the genus’ members, the two sub-Saharan species *Acanthodactylus* (*A. guineensis, A. boueti*) species are not desert/arid-adapted lizards. We visualized this simply by plotting annual mean temperature and precipitation (Fig. 4a). As a next step we used the Maxent algorithm^46^ to predict the species’ potential distribution range and to determine which environmental factors describe the distribution the best.

**Figure 5:**
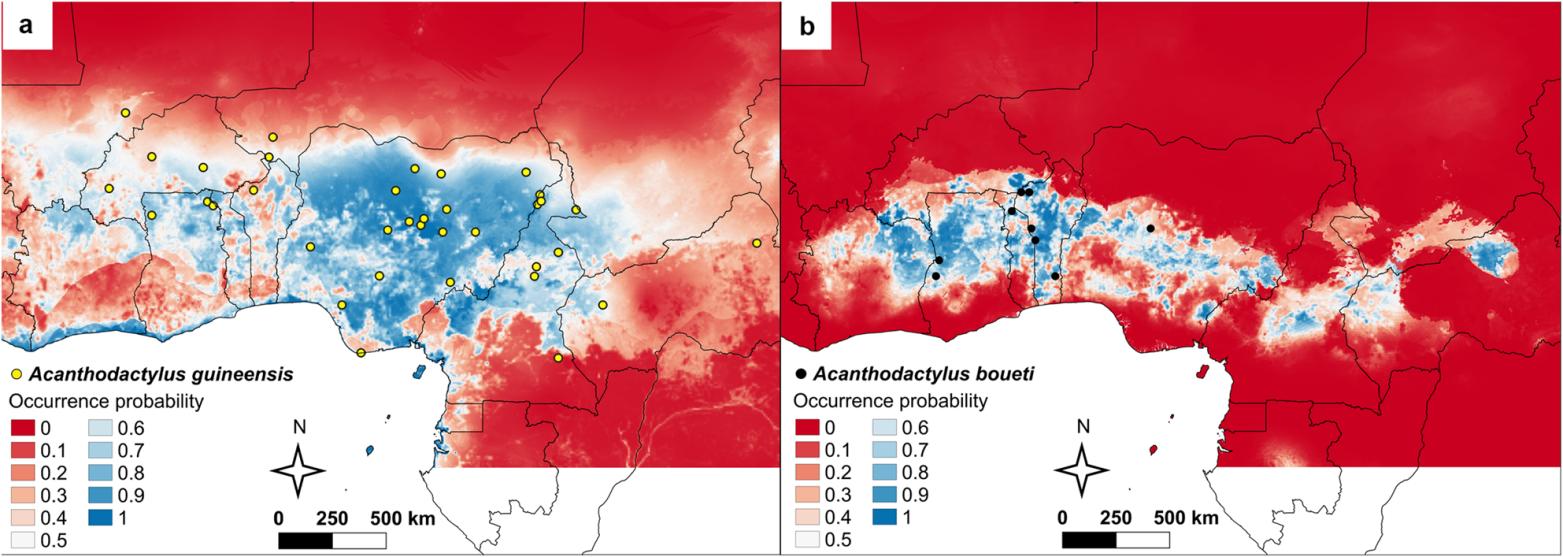
(**a**,**b**) Maxent model showing occurrence probabilities (higher with colder colors) of *A. guineensis* (**a**) and *A. boueti* (**b**) based on environmental parameters (i.e., Grinnellian niche). Areas in white were not modeled. The figure was created using QGIS 3.14.16 (https://www.qgis.org).

The *Acanthodactylus guineensis* model performed well and the current range was predicted with higher accuracy than simply by chance (mean AUC = 0.842 ± 0.045). The populations in southern Burkina Faso, northern Ghana, Nigeria, northern Cameroon and western Central African Republic were predicted with high occurrence probability (Fig. 5a, Supplementary Fig. S5a online). In addition, there is a high probability that *A. guineensis* finds suitable abiotic conditions in northwestern and central Benin, northern and central Togo, and northern Côte d’Ivoire. On the other hand, the populations in northeastern Benin, southern Niger, southern Cameroon, and particularly eastern Central African Republic appear to inhabit less suitable habitats according to our model.

The environmental variable with the highest contribution (68.2%) to the final model was ‘Potential Evapotranspiration of the Wettest Quarter’ (PETwet)—*A. guineensis* has higher occurrence probability in areas with low potential evapotranspiration. The second most important variable was forest cover (contributing 22.6% to the final model) with decreasing occurrence probability linked to increasing forest cover. Another 4.9% to the model are contributed by ‘Precipitation of the wettest week’ (bio13) with increasing occurrence probability in areas with higher rainfall (Supplementary Figs. S6, S7 online). The model thus confirms that the species has a certain dependence on humidity and rainfall.

The Maxent model for *A. boueti* achieved even higher accuracy than the *A. guineensis* model (mean AUC = 0.977 ± 0.006). The best predictors are predominantly linked to rainfall and comprised ‘Precipitation of coldest quarter’ (bio19, 32.7%, positively correlated with occurrence probability), ‘Precipitation of driest week’ (bio14, 20.6%, negatively correlated with occurrence probability) and ‘Mean temperature of coldest quarter’ (bio11, 13.2%, positively correlated with occurrence probability). The inferred potential distribution comprised Côte d’Ivoire in the West, and central Nigeria, central Cameroon and parts of Central African Republic (Fig. 5b, Supplementary Figs. S5b, S6, S7 online).

## Discussion

Our study contributes to the understanding of the evolution of lacertid lizards by reliably resolving the phylogenetic placement of a biogeographically enigmatic taxon, the sub-Saharan *A. guineensis*, based on a well-supported mitogenomic phylogeny.

The approach using a shotgun sequencing strategy on preserved museum specimens to assemble whole mitogenomes proved successful and our phylogenetic reconstruction placed *A. guineensis* into the Western clade of *Acanthodactylus*, but not in the hypothesized position as sister to the entire genus. For the first time, we can reliably confirm previous hypotheses based either purely on morphological data^17, 18^ or limited genetic data (i.e., only two mitochondrial fragments) that *A. guineensis* indeed belongs to the genus *Acanthodactylus*, a finding that is essential for reconstructing the phylogenetic and paleoclimatic history and deepening our understanding of the evolution of that genus. Previous molecular phylogenies that contained the only other *A. guineensis* specimen (A275^6, 14^) have found the species at very disparate places on the lacertid phylogeny, sometimes not within *Acanthodactylus* at all^6^. The reliable placement of this taxon within the family of Lacertidae was therefore a priority in lacertid systematics and constitutes one of the key outcomes of our study.

Considering the new results, an African origin of the genus *Acanthodactylus* is still the most likely biogeographical hypothesis^14^, with independent expansions of two (Western and Eastern) of the three main clades into the Middle East (and east further into Asia in the case of the Eastern clade).

Morphologically, all examined individuals of *A. guineensis* match the detailed first description^40^ and those of the two known synonyms *Eremias (Taenieremias) benuensis*^47^ and *Eremias mandjarum*^48^ with their rather unique combination of morphological features such as the nasal scale arrangement, the toe fringes, the almost coadunate collar and the absence of an occipital scale (Supplementary Material online). Minor morphological variation (e.g., in the number of scales under the 4th toe, number of prefrontals and supraciliaries) was detected (especially in individual JFT 4143 which even has a minute occipital granule, a feature known also for *A. boueti*).

*Acanthodactylus guineensis* currently occupies a relatively large distribution range for a lacertid lizard, spanning almost 3000 km (around 2,000,000 km^2^) from Burkina Faso in the West to the eastern Central African Republic (Fig. 3;^45^). Our newly detected specimens in the MfN collection, including the new synonym *Eremias mandjarum* Sternfeld, 1916, and the record from the Pendjari National Park in Benin^38^, close previously apparent range gaps between the easternmost record and the Cameroonian records and between Ghana and Nigeria (although *A. guineensis* records from Togo are still missing). The elevational range the species occupies from sea level to 1300 m is quite large, and there is a distinct gap between 900 and 1000 m where no individual was recorded (Fig. 4b). Whether this is a sampling artifact or reflects the reality requires increased sampling effort in such areas. Only limited information on microhabitat and substrate use is available for *A. guineensis* from the literature (nothing appears to be known for *A. boueti*), but the current notion is that *A. guineensis* predominantly inhabits sandy habitats, often along rivers^43–45^. This is in agreement with the presence of toe fringes in *A. guineensis*, similar to all other *Acanthodactylus* spp. Nevertheless, *A. guineensis* has also occasionally been recorded from harder soil and rocky habitat structures^43^; JFT pers. obs.). Although Meinig and Böhme^44^ did not find morphological variation among *A. guineensis* along an East–West transect that would allow recognition of subspecies, their dataset did not include the specimens from Central African Republic. Our extended dataset including the latter, underpinned with climate, altitude and additional (macro-) habitat data provide at least some indication that there is still hidden diversity among these lizards. Additional in-depth assessment of *A. guineensis* (and *A. boueti*) taxonomy is consequently still required.

Our new phylogeny combined with environmental niche models suggests that the only two sub-Saharan species of the genus (*A. guineensis*, *A. boueti*) are not sister taxa despite occupying roughly similar niches in precipitation-rich and hot tropical environments, opposed to the mainly xeric adapted remaining 42 species of *Acanthodactylus*. It is worth noting that the phylogenetic position of *A. boueti* is not fully concordant among the phylogeny presented here and another published tree^6^ calculated with a very similar data set for these taxa, as no phylogenomic backbone data for relationships within *Acanthodactylus* was available. In fact the previous studies were based on the same individual of *A. boueti,* and largely on the same sequences (partial sequences of the mitochondrial cytochrome b and 12S rRNA genes, plus the nuclear c-mos gene)^6, 14^ as herein. The added c-mos sequence may be responsible for a slightly different position of *A. boueti* in previous studies, where it was placed sister to the Western clade, while our mitochondrial gene tree places it in a nested position within this clade. In any case, a possible sister-group relationship *A. boueti* and *A. guineensis* has not been recovered in any species or gene tree reconstruction to date^6, 14^. Although for a full clarification of its relationships more extensive, preferably phylogenomic data sets are necessary, we consider it as very unlikely that *A. guineensis* and *A. boueti* are closely related to each other given their high genetic distance in the genes analysed^14^.

Looking at a larger scale, the tropical habitat makes *A. guineensis* (and *A. boueti*) rather unique among the entire Northern arid clade (as opposed to its sister, the predominantly tropical Equatorial African-Arabian clade). In the otherwise robust molecular timetree generated by Garcia-Porta et al.^6^ *A. guineensis* had unresolved relationships and was sometimes even recovered as basal to the entire Northern arid clade^6^, but only two mitochondrial genes of one individual of *A. guineensis* were available for that study.

Inferring a timetree from a purely mitochondrial phylogeny as provided herein, without inclusion of nuclear DNA sequences, would likely result in overestimated divergence times especially for old nodes^49^. We therefore did not reconstruct a new time-calibrated phylogeny, but for discussion rather rely on diversification dates from the phylogenomic analysis of Garcia-Porta et al.^6^. Because our analysis places *A. guineensis* within the *Acanthodactylus* crown group, it is unlikely that the timing of the crown group diversification of the genus during the tropical Eocene epoch (36.7 mya) recovered by Garcia-Porta et al.^6^ will be strongly affected by the improved phylogenetic position of this species.

Within the Saharo-Eurasian clade of Eremiadini, the available phylogenomic evidence supports a subclade with *Acanthodactylus* and *Mesalina* (the Northern arid clade) and a second subclade with *Adolfus, Holaspis,* and *Omanosaura* (the Equatorial African-Arabian clade)^6^. However, the relationships of several other genera in the Saharo-Eurasian clade remains largely unresolved. *Congolacerta* and *Gastropholis* appear to belong to the Equatorial African-Arabian clade, and *Eremias* and *Ophisops* to the Northern arid clade^6^ but the exact phylogenetic placement of these genera are poorly supported^50^. Our mitogenomic tree, in agreement with another study^50^ places the purely arid-adapted *Mesalina* sister to *Acanthodactylus* (including *A. guineensis*), and the equally arid-adapted *Eremias* sister to this clade. This topology, with *Acanthodactylus* nested within the arid-adapted genera and *A. guineensis* furthermore not being sister to all other species of its genus, suggests adaptation to arid conditions was ancestral for *Acanthodactylus,* and the ancestor of *A. guineensis* colonized very early on, maybe before the Eocene–Oligocene extinction event (~ 33.7 MYA), the woodland and forest/open habitat ecotones it inhabits today. The very long branch connecting *A. guineensis* to its sister group (only comparable in length to that of the other sub-Saharan species *A. boueti*) suggests that this species represents a relic lineage that may have persisted for a long time without diversification, or, alternatively, has experienced severe extinctions along the branch.

In this scenario, the drastic change in climatic conditions from Eocene to Oligocene then triggered the extensive diversification of arid-adapted *Acanthodactylus*. The second tropical *Acanthodactylus* with sub-Saharan distribution—*A. boueti*—albeit occupying similar environmental conditions as *A. guineensis,* likely entered humid tropical environments independently at a later time, as indicated by its rather distant relationship to *A. guineensis* (Supplementary Fig. S2 online) in combination with its estimated divergence time within the Western clade of *Acanthodactylus* after the Eocene–Oligocene extinction event at between 29.7 MYA^6^ and 17.8 MYA^14^.

However, considering the poorly resolved relationships among several genera in the Saharo-Eurasian clade, an alternative hypothesis should not be completely discarded: the adaptation of *A. guineensis* to humid biomes may represent an ancestral condition of the genus or the Northern arid clade, and several colonization events and radiations into more arid biomes may have taken place. Given the current phylogenetic knowledge this scenario is clearly not parsimonious, but it should be reconsidered if future phylogenomic studies would support a split of *Acanthodactylus* from a more basal node in the Northern arid clade, and/or an (unlikely) phylogenetic position of *A. guineensis* sister to all other *Acanthodactylus*.

The Maxent niche models and the temperature-precipitation regression (Fig. 4a) support our hypothesis that *A. guineensis* and *A. boueti* constitute tropical species. For both species, occurrence probability rises with increasing values for environmental parameters linked with humidity or direct rainfall (or decreasing in the case of potential evapotranspiration). Nevertheless, while being tropical species both appear to be open habitat and not forest lizards^43, 45^ and only briefly enter more densely vegetated areas as indicated by our habitat analyses and the decreasing occurrence probability with an increase in forest cover in the case of *A. guineensis* (Supplementary Figs. S6, S7 online).

The transitional period from the greenhouse climate of the early Eocene to the cooler and drier Oligocene is considered the most significant interval in Earth history since the extinction of the dinosaurs^51^. Major species turnovers have been documented as a result of the Eocene–Oligocene extinction event, both on land (e.g., for plants, mammals, lizards, turtles) and in marine environments^52–54^. The continuous climatic cooling and drying since the Eocene climatic optimum further resulted in changes in vegetation, for example, in Europe, Asia and North America, dense Eocene forests were replaced by more open country^52^. In marine environments, especially the colder winter temperatures—a climate parameter which can impact terrestrial ectothermic lizards greatly as species might be required to adopt a hibernation period to survive unsuitable cold periods—have been determined to being largely influential in facilitating faunal turnover during the Eocene–Oligocene transitional period^53^. We here present another example where the major climatic changes from the tropical Eocene to the cooler and drier Oligocene had an impact in shaping diversity, distribution, and molecular evolution of reptiles, in this case of the predominantly Palearctic lizard genus *Acanthodactylus*.

To conclude, our molecular results demonstrate that despite some divergent morphological features compared to other *Acanthodactylus*, *A. guineensis* is certainly a member of this genus. We wish to stress, however, that the genetic material analyzed was not from a name-giving type specimen. *Acanthodactylus guineensis* appears to have remained in tropical habitat from periods prior to the Eocene–Oligocene transitional period ~ 33.7 MYA, while the major *Acanthodactylus* radiation into arid environments happened after the mass extinction event accompanied by climatic changes from tropical to cooler and drier environments. *Acanthodactylus boueti* likely re-entered tropical environments independently at a later period of time. While *A. guineensis* is clearly not a desert taxon like the majority of *Acanthodactylus* species, it nevertheless seems to inhabit mainly sand habitats even within its precipitation-rich environment. The preference for sandy substrate explains the toe-fringes typical for the genus^43–45^, even though in *A. guineensis* they are poorly developed. The rather unexpected report of *A. guineensis* also inhabiting harder soil and rocky habitat structure^43–45^ in combination with some morphological variation we detected and a possible gap in elevational distribution corroborate the necessity of increased sampling effort and in-depth molecular analyses of the species using whole genomes or at least nuclear markers.

## Methods

The following museum acronyms are used (mostly following^55^):

BMNH, NHM, BM: Natural History Museum, London, formerly British Museum of Natural History (UK);

CAS: California Academy of Sciences, San Francisco (USA);

MHNC: Musée d’histoire naturelle, La Chaux-de-Fonds (CH);

MHNG: Muséum d’Histoire Naturelle, Genève (CH);

MNHN: Muséum national d’Histoire naturelle, Paris (F);

SMNS: Staatliches Museum für Naturkunde, Stuttgart (D);

UWBM The Washington State Museum of Natural History and Culture/Burke Museum, University of Washington (USA);

ZMB: Museum für Naturkunde Berlin, formerly Zoologisches Museum Berlin (D).

### Study organism and taxonomic background

*Acanthodactylus* was first described as a subgenus of *Lacerta* Cuvier (sic)^56^, the type species is *A. boskianus* (Daudin, 1802). The few meristic characters described to be diagnostic for *Acanthodactylus*^56^ include: collar connected in the center but free on the sides, tempora squamosal, i.e., temporal region covered by small scales rather than larger shields, ventral scales rectangular and arranged in longitudinal rows, digits acutely fimbriate-denticulate forming “toe fringes”. Fringed toes have evolved in various shapes multiple times in lizards and in Lacertidae, and when triangular, projectional or conical as in *Acanthodactylus* they are commonly seen as adaptation to windblown sand substrate^57^.

The enigmatic *Acanthodactylus guineensis* is among the lesser-known species of the Lacertidae. Only limited information is available regarding the species’ morphology, habitat and distribution (see Meinig & Böhme^44^ for a review and references therein; and ^34, 38, 45, 58^). Based on one young specimen, *A*. *guineensis* was described as member of the genus *Eremias* Fitzinger, 1834 by Boulenger^40^. Boulenger does not mention the slightly projecting third row of scales around the toes and fingers (“fringed toes”) of the type specimen in his quite comprehensive description^40^, probably because he did not notice it in such a small (SVL 24 mm) specimen and because the projection is indeed rather minor compared to other members of the genus. This is probably also the reason why he did not place *guineensis* in the genus *Acanthodactylus* Wiegmann, 1834 but in *Eremias* Fitzinger, 1834 (in^56^).

About 30 years later, Boulenger revised the genus *Eremias* and divided it into five sections he assumed to be natural associations^59^: (1) *Taenieremias* Boulenger, 1918—monotypic, type species *Eremias guineensis* Boulenger, 1887 (currently *Acanthodactylus guineensis*); (2) *Lampreremias* Boulenger, 1918—type species *Eremias nitida* Günther, 1872 (currently *Heliobolus nitidus*); (3) *Pseuderemias* Boettger, 1883—type species *Eremias mucronata* (Blandford, 1870) (currently *Pseuderemias mucronata*); (4) *Mesalina* Gray, 1838—type species *Eremias rubropunctata* (Lichtenstein, 1823) (currently *Mesalina rubropunctata*); (5) *Eremias* s. str. Fitzinger, 1834—type *Eremias velox* (Pallas, 1771) (currently *Eremias velox*).

Monard^47^ described *Eremias* (*Taenieremias*) *benuensis* from Cameroon based on a few minor morphological differences compared to *E. guineensis*, but in 1969 *E. benuensis* was synonymized with *E. guineensis*^60^.

Salvador^17^ and one year later also Arnold^18^ in their respective major revisions of the genus *Acanthodactylus* both found that *Eremias* (*Taenieremias*) *guineensis* agrees with all the characteristic morphological features of *Acanthodactylus* with the exception of the arrangement of scales around the nostril. However, it was suggested that the *E. guineensis* condition with an extra suture across the area occupied by the first upper labial scale to produce a smaller first upper labial is easily derived from that found in *Acanthodactylus,* evidenced by BMNH 1966.430, a juvenile *A. erythrurus lineomaculatus* (sic) with a similar scale arrangement^18^. Within *Acanthodactylus*, both authors placed *guineensis* in the Western clade and in the *Acanthodactylus erythrurus* group which was assumed to consist of *A. erythrurus, A. savignyi, A. boueti, A. guineensis*, and *A. blanci* which was considered a subspecies of *A. savignyi* at the time^14, 20^.

### Molecular data and phylogenetic analyses

We aimed at reconstructing a well-supported phylogeny of the genus *Acanthodactylus* using whole mitochondrial DNA sequences, assembled by means of a shotgun next generation sequencing strategy. Analyses of whole mitogenomes have been shown to resolve many nodes of the lacertid tree with high statistical support (e.g.^31^). We retrieved muscle tissue from a museum voucher of *A. guineensis* (ZFMK 59511 from Daroha, near Bobo Dioulasso, Burkina Faso^43^) that likely was never in contact with formalin for preservation and therefore offered good chances to obtain sufficient amounts of DNA of decent quality for sequencing. We extracted genomic DNA using the Qiagen DNeasy Blood and Tissue Kit (Qiagen, Hilden, Germany) following the protocol provided by the manufacturer. We additionally sequenced a tissue sample of a freshly caught individual of *Acanthodactylus schmidti* (SB 642) from Abu Dhabi (UAE) to increase sampling of *Acanthodactylus* spp. in the mitogenomic tree. The lizard was euthanized by injection of an aqueous solution of benzocaine (20%) into the body cavity. Subsequently, a sample of muscle tissue was taken from the thigh, preserved in 98% Ethanol and stored in − 80 °C. Handling, euthanizing and collection of tissue samples of *A. schmidti* individuals was approved by the NYUAD Institutional Animal Care and Use Committee (IACUC 19-0002) and UAE No Objection Certificate (NOC 8416), and all applied methods were performed in accordance with the relevant guidelines and regulations.

Genomic DNA of SB 642 was extracted from ethanol-preserved muscle tissue using the Qiagen MagAttract HMW DNA Kit (Qiagen, Hilden, Germany) for high molecular weight DNA. We determined DNA yields on a Qubit fluorometer (Qubit, London, UK) with a dsDNA high sensitivity kit. Totals of 52 ng and 80 ng of DNA (diluted in 26 µl 10 mM Tris·Cl, 0.5 mM EDTA, pH 9.0 (AE buffer)) from *A. guineensis* and *A. schmidti*, respectively, were used for library preparation.

ZFMK 59511 libraries were prepared with NEB Ultra II FS DNA (New England Biolabs, Ipswich, MA, USA) kit as per protocol instructions with input below 100 ng. For sample SB642, linked reads were generated on a 10X Genomics Chromium following Genome reagent kits v2 instructions. Resulting libraries’ concentration, size distribution and quality were assessed on a Qubit fluorometer (Qubit, London, UK) with a dsDNA high sensitivity kit and on an Agilent 2100 Bioanalyzer (Agilent, Santa Clara, CA, USA) using a High Sensitivity DNA kit. Subsequently, libraries were normalized and pooled, and pools quantified with a KAPA Library quantification kit for Illumina platforms (Roche Sequencing, Pleasanton, CA, USA) on a ABI StepOnePlus qPCR machine (Thermo Fisher Scientific Inc., Waltham, MA, USA), then loaded on a SP flowcell and paired-end sequenced (2 × 150 bp) on an Illumina NovaSeq 6000 next generation sequencer (Illumina, San Diego, CA, USA), and a S2 flowcell for linked read library. Raw reads were deposited in the Sequence Read Archive (SRA) under BioProject ID PRJNA700414 . All mitogenome assemblies and original alignments were deposited in Figshare under  https://doi.org/10.6084/m9.figshare.13754083.v1 .

Raw FASTQ sequenced reads were first assessed for quality using FastQC v0.11.5^61^. The reads where then passed through Trimmomatic v0.36^62^ (parameters ILLUMINACLIP: trimmomatic_adapter.fa:2:30:10 TRAILING:3 LEADING:3 SLIDINGWINDOW:4:15 MINLEN:36) for quality trimming and adapter sequence removal. Following the quality trimming, the reads were assessed again using FastQC. The executed workflows were performed using BioSAILs^63^.

For SB 642 (*A. schmidti*), we received 436,370,000 single-end reads (average read length 139.5 bases (b)) with a raw coverage of 29.29X and a scaffold N50 of 21.12 kilobases (kb). For the MITObim analysis, read number for SB 642 was randomly reduced to 1,000,000 reads (360,463,035 b) using the *awk* command. For ZFMK 59511 (*A. guineensis*), we received 57,936,345 paired-end reads (average read length 151 b, raw coverage ~ 10x). Subsequently, the two sets of reads were converted to interleaved format. We used the quality filtered reads to assemble the mitogenomes of A. *guineensis* and *A. schmidti* using an iterative mapping strategy in MITObim v. 1.9.1^64^. We used the *Acanthodactylus aureus* mitogenome (GB accession number xxxxx; assembly method is provided in the next paragraph) as seed for both samples; this rendered an initial mapping of a conserved region from a more distantly related individual unnecessary^64^. We therefore applied the –quick option in MITObim and iterations were run until no additional reads could be incorporated into the assembly (14 in *A. guineensis*, eight in *A. schmidti*).

We also assembled complete or nearly complete mitogenomes for additional twelve species of Gallotiinae and Eremiadini using anchored hybrid enrichment sequence data from Garcia-Porta et al.^6^ (see Supplementary methods in^6^ for extraction protocol and sequencing methods and Supplementary Table S1 online in^6^) with the *Podarcis muralis* complete mitogenome (GB accession number NC_011607) as reference sequence. The raw data of each individual was quality filtered using Trimmomatic v0.36^62^ (parameter MINLEN:45) and assembled using MITObim v. 1.9.1. All multiple alignment files generated in the final MITObim iteration were imported to Geneious R11 (https://www.geneious.com) to check for assembly quality and coverage.

The resulting assemblies were annotated with MITOS^65^ using defaults settings with the vertebrata database as a reference. The annotated assemblies were imported into PhyloSuite^66^ together with existing mitogenomic sequences of Lacertidae, and *Blanus cinereus* (Amphisbaenia; outgroup) available in GenBank (as of July 2020). Details of all mitogenomic sequences included in this study and the corresponding GenBank accession numbers are provided in Supplementary Table S1 online. Using PhyloSuite, we exported all protein coding sequences plus the two rRNAs. The resulting files were aligned with MAFFT^67^ using “auto” settings. Alignments of coding sequences were refined with MACSE^68^ to account for open reading frame structure during the alignments. The protein coding and rRNAs alignments were finally concatenated into a single alignment delimiting partitions by marker and, for the protein-coding genes, by codons within markers. Mitogenome phylogenies were inferred using a maximum likelihood approach with IQ-TREE v. 1.5.4 software^69^ and Bayesian inference (BI) analysis with MrBayes 3.2^70^.

For the maximum likelihood approach, best-fitting partitioning schemes and substitution models were selected based on the Akaike Information Criterion (AIC) using the heuristic algorithms implemented in the MFP + MERGE option (Supplementary Table S3 online). After ML inference, branch support was assessed with 1000 ultrafast bootstrap replicates. As the alignments used for phylogenetic inference contained more than one sequence for some species, these terminals in the resulting tree were collapsed a-posteriori for aesthetic reasons using FigTree v1.4 (as depicted in Fig. 1). An uncollapsed version of this tree is presented in Supplementary Fig. S1 online. For the BI analysis, the best-fitting partition/substitution model scheme, as selected by the ModelFinder algorithm in iQTree (Supplementary Table S4 online), was implemented with MrBayes 3.2^70^. Results of two independent runs of 10 million generations, each comprising four Markov Chains (three heated and one cold), were sampled every 1000 generation. Chain mixing and stationery was assessed by examining the standard deviation of split frequencies and by plotting the –lnL per generation using Tracer 1.5 software^71^. Samples corresponding to the initial phase of the Markov chain (25%) were discarded as burn-in and the remaining results were combined to obtain a majority rule consensus tree and the respective posterior probabilities of nodes.

An additional, more comprehensive, alignment was produced containing all currently known *Acanthodactylus* species to obtain a higher resolution perspective on the phylogenetic placement of the target species. Since no nuclear data was available for *A. guineensis*, we extracted from the newly obtained sequences the four best represented mitochondrial gene fragments across Lacertidae, as compiled by Garcia-Porta et al.^6^, despite the known shortcomings of partial mitochondrial datasets to resolve lacertid relationships^9, 72, 73^. The corresponding sequences of the ribosomal RNAs (12S, 16S), COB, and NADH-dehydrogenase subunit (ND4) were later added to the alignment of^6^ using *–add* and *–keeplength* options in MAFFT (Supplementary Table S5 online).

### Species distribution modeling

As basis for characterizing the species’ bioclimatic envelopes, we compiled an updated distribution map for *A. guineensis* and *A. boueti* including new discoveries in museum collections from the current study (see Supplementary Material online), all known records from the literature^16, 34, 37, 38, 40, 42, 44, 45, 47, 58, 60, 74–77^ and from the Global Biodiversity Information Facility (GBIF^78^). We provide all coordinates as latitude (decimal degrees), longitude (decimal degrees). Due to the general scarcity of records for *A. guineensis* and *A. boueti*, we added two localities for *A. guineensis* (12.5°, − 2.5°; 7.5°, 13.5°) and one locality for *A. boueti* (− 2.5°, 7.5°) published in Trape et al.^45^ that were not found on GBIF or in other publications, by extracting the center coordinates of the respective 1 × 1 degree grid. These additional localities have coordinates with very low accuracy and the corresponding environmental variables extracted from a 1 × 1 km resolution grid (30 arc-seconds; see below) therefore have high degrees of uncertainty. When examined, the majority of values fell within the total range for the respective environmental variable (the two exceptions are mentioned in the Results section), we consequently decided to keep them for our analysis. We reduced the final locality dataset to one record per km^2^ (the resolution of the environmental input data, see below) to avoid pseudoreplication using the R package spThin^79^. We then tested whether the remaining presence points are randomly dispersed or clustered using the Nearest Neighbor Index NNI in the R packages sp^80^ and spatialEco^81^. NNI was 1.4 for *A. guineensis* and 1.2 for *A. boueti*, indicating that the filtered point dataset consists of randomly distributed points which are not spatially autocorrelated.

As environmental parameters we downloaded spatial layers of the Terrestrial Ecoregions of the World (TEOW)^82^, global bioclimate and elevation layers^39^, data on potential evapotranspiration^83^ and global aridity index^84^ with a spatial resolution of 30 arc-seconds (~ 1 km^2^ near the equator). Although the climate datasets are interpolated from data from weather stations much farther apart from one another and are consequently not measurements of actual environmental conditions, they were rigorously cross-validated with observed data (including satellite data) during the development of the dataset and generally showed high correspondence with observations (especially temperature variables)^39^. Since for many applications such as our species distribution models data at high spatial resolution (i.e., 1 × 1 km) are preferable over lower resolution to capture variation for example across steep climate gradients in mountains^39^ we chose the 30 arc-seconds dataset over the 5 arc-minutes dataset (~ 9 × 9 km near the equator) for the ecoregions and climate datasets. In addition, we downloaded spatial layers with forest and grassland/scrub/woodland cover from the harmonized soil database (only available in 5 arc-minutes spatial resolution^85^). We downscaled the forest and grassland/scrub/woodland dataset to 30 arc-seconds resolution despite the resulting slight inaccuracy, which we kept in mind during interpretation but did not consider relevant for the vegetation datasets.

In order to compare the prevailing climate within the distribution range of *A. guineensis* and *A. boueti* to all other species of the genus *Acanthodactylus* we downloaded all available records of the other currently known *Acanthodactylus* species from GBIF. We cleaned the downloaded datasheet by deleting all entries with missing data for either latitude, longitude, species epithet, or all of those, and removed duplicates or spelling mistakes. We further removed all GBIF entries with imprecise coordinates, i.e. records with coordinates that when plotted landed just outside of coastal areas in the ocean instead of on land (usually coordinates with only two decimals). In addition, we added several curated locality data from Garcia-Porta et al.^6^ as available from Figshare (https://doi.org/10.6084/m9.figshare.8866271.v1/). Following Tamar et al.^14^ we treated *A. lineomaculatus* as a junior synonym of *A. erythrurus* and changed the respective records in our database accordingly. For species that did not have records published on GBIF we added further locality data from the literature (Supplementary Table S6 online). The final database contained 4286 records from all 44 currently recognized species of *Acanthodactylus*. We acknowledge that our database is by no means complete, however, we wanted to follow the most conservative approach and use only records that are supported by a voucher specimen and can be traced back using published databases. We trust that our record list covers a nearly complete representation of the ecological conditions inhabited by all currently accepted *Acanthodactylus* species.

Using the bioclim dataset^39^ we plotted annual mean temperature (bio1) and annual precipitation (bio12) for all locality records in our *Acanthodactylus* species database and compared the respective data for *A. guineensis* and *A. boueti* with its congeners. We developed species distribution models using Maxent 3.4.1^46^ for *A. guineensis* and *A. boueti*. Maxent applies the maximum entropy principle^86^ for model fitting under the basic premise that the estimated species distribution deviates from a uniform distribution as minimally as required to explain the observations^46^. We used the cleaned datasets of *A. guineensis* and *A. boueti* with only one record per km^2^ as input presence locality data. We extracted environmental data for all presence sites from the 19 bioclim variables (bio1-19), elevation (alt), aridity index (AI), percentage cover of forest (forest) and grassland, scrub and woodland (grass) per grid cell, as well as four parameters comprising potential evapotranspiration (PET): PET of the wettest, driest, warmest and coldest quarter of the year (PETwet, PETdry, PETwarm, PETcold). To avoid issues resulting from correlated parameters we reduced the number of environmental predictors by performing multicollinearity analyses using the Variance Inflation Factor (VIF) with a threshold < 10 for non-collinearity^87^ in the R package usdm^88^. The VIF, one of the most commonly used factors in collinearity analyses, is the result from regressing the predictor variable against all other predictor variables. The VIF measures how strongly each predictor can be explained by the rest of predictors^89^. After removing all variables with collinearity problems our final environmental input variable dataset comprised bio3, bio11, bio13, bio14, bio18, bio19, PETcold, PETdry, PETwet, forest, grass. We ran the Maxent algorithm with the following settings: we chose 10,000 random background points from a rectangular area extending an additional 500 km in every compass direction from the outermost presence sites of *A. guineensis* for both species^90^, allowed only linear and quadratic features and chose the output format cloglog. For *A. guineensis* we had 38 presence sites, of which we used 70% for model training and 30% for testing with 25 model replicates of type "subsampling" and a regularization multiplier of 2.5^91^. For *A. boueti*, due to low number of presence sites (N = 11), we did not use a training dataset but ran the model with eleven replicates and bootstrapping (following Morales et al.^91^) and a regularization multiplier of 1. The predictive model performance was evaluated using the area under the receiver operating curve (AUC) which measures the ability of predictions to discriminate between presences and absences, irrespective of the absolute value of the predictions^92^. Finally, we plotted the median of all models to predict the potential range of each species and determined the most important environmental predictors.

## Supplementary Information

Supplementary Information.
