# Supplementary material for: Mitogenome analyses elucidate the evolutionary relationships of a probable Eocene wet tropics relic in the xerophile lizard genus Acanthodactylus
Source: Sci Rep. 2021 Mar 1;11:4858. doi: 10.1038/s41598-021-83422-7 (PMC7921649; doi:10.1038/s41598-021-83422-7)
Supplement: Supplementary file 1 — Supplementary Information. [file 41598_2021_83422_MOESM1_ESM.pdf]

Mitogenome analyses elucidate the evolutionary relationships of a probable Eocene wet tropics relic in the xerophile lizard genus *Acanthodactylus*

Sebastian Kirchhof[1*], Mariana L. Lyra[2], Ariel Rodríguez[3], Ivan Ineich[4], Johannes Müller[5], Mark-Oliver Rödel[5], Jean-Francois Trape[6], Miguel Vences[7], Stephane Boissinot[1]

[1]New York University Abu Dhabi, Saadiyat Island, Abu Dhabi, United Arab Emirates
[2]Universidade Estadual Paulista, Instituto de Biociências, Departamento de Biodiversidade and Centro de Aquicultura (CAUNESP), Rio Claro, SP, CEP 13506–900, Brazil
[3]University of Veterinary Medicine of Hannover, Institute of Zoology, Bünteweg 17, 30559 Hannover, Germany
[4]Institut de Systématique, Évolution, Biodiversité (ISYEB), Muséum national d'Histoire naturelle, CNRS, Sorbonne Université, École Pratique des Hautes Études, Université des Antilles, CP 30, 57 rue Cuvier, 75005 Paris, France
[5]Museum für Naturkunde, Leibniz Institute for Evolution and Biodiversity Science, Invalidenstr. 43, 10115 Berlin, Germany
[6]Laboratoire de Paludologie et Zoologie tropicale, UMR MIVEGEC, B. P. 1386, Dakar, Senegal
[7]Technische Universität Braunschweig, Zoological Institute, Mendelssohnstr. 4, 38106 Braunschweig, Germany

*Author of correspondence: sebastian.kirchhof@nyu.edu

Supplementary material

Supplementary methods

<u>Morphological analyses and holotype redescription of a new synonym</u>

Acanthodactylus guineensis shows morphological differences to other *Acanthodactylus* species leading to frequent misidentification of specimens. In order to update the distribution range and obtain baseline data for species distribution modeling we examined museum vouchers of Lacertidae from Central and West Africa, focusing on *A. guineensis* and its synonyms, as well as on unlabeled museum specimens which superficially resembled *A. guineensis*. Additionally, we examined specimens of other species of *Acanthodactylus,* as well as *Latastia* spp. and *Heliobolus* spp. We investigated material from the collections of the Museum für Naturkunde Berlin (ZMB); Muséum national d'Histoire naturelle, Paris (MNHN); Staatliches Museum für Naturkunde, Stuttgart (SMNS); The Washington State Museum of Natural History and Culture/Burke Museum, University of Washington (UWBM); and a specimen collected by Jean-François Trape (JFT). Specimens were determined using the original descriptions of the type material.
For new specimens we examined the following meristic and mensural characters: Snout-vent-length (SVL); tail length; head length (from the tip of the snout to the

posterior side of the tympanum); pileus length (from the tip of the snout to the medial posterior edge of the parietals); forelimb length (ventrally from its conjunction with the trunk to the tip of the 4th finger); hind limb length (ventrally from its conjunction with the trunk to the tip of the 4th toe); number of transverse ventral scale rows from the collar to the preanal scales; number of longitudinal ventral scale rows; number of dorsal scale rows at midbody; number of supralabials anterior to the subocular; position and number of nasal scales; presence and shape of tympanic shield; presence of auricular denticulation; presence of occipital scale; number, position and size of frontonasal, prefrontals, frontal, parietals, interparietals, loreals, supraoculars, supraciliaries; number of enlarged scales and granules surrounding the large supraoculars (we counted clearly enlarged scales and all granules posterior and anterior the supraoclulars separately excluding granules in contact with the supraciliaries which were counted independently); number and condition of collar scales; number of chin shields; number of scales under the 4th toe; number of rows of scales around 4th finger and 4th toe; number and position of femoral pores. All mensural characters were measured using a digital caliper to 0.1 mm.

Supplementary results

Specimen (ZMB 25479) was originally labeled *Eremias* n. spec., Typ., Uam, Houy. Later, the information had been extended to *Eremias mandjarum* * (*indicating type status) Sternfeld, 1916, Uam, Houy. The specimen was collected by Robert Houy on 3 March 1903 in "Neukamerun" (New Cameroon), a former French colonial territory which later also belonged to Germany (1911-1916). Today, the territory is part of several countries: Chad, Central African Republic, Republic of the Congo, and Gabon. The type locality, the Ouham River, originates between the prefectures Nana-Mambéré and Ouham-Pendé (Central African Republic) and joins the Chari River in Chad. The part of the Ouham River running through Chad, however, was not part of New Cameroon, consequently the type locality has to be regarded as located in today's Central African Republic, and not eastern Cameroon[1,2].
We provide below a detailed description of specimen ZMB 25479. We add the values from Sternfeld's original description[3] in square brackets. Values for symmetric characters are given as left/right unless they are the same on both sides.

<u>Re-description of the holotype of *Eremias mandjarum* Sternfeld, 1916 (ZMB 25479)</u>

Type locality: Ouham River ["Uamfluß"], Central African Republic (no detailed coordinates are known, we assigned the coordinates of the town Bozoum to the specimen: 6.30°, 16.37°).
Adult female; snout-vent-length 57 mm [57]; tail 97 mm [97]; head length 13.5 mm [13.5]; pileus length 12.7 mm; forelimb length 18 mm [18]; hind limb length 29 mm [29]; 31 [31] transverse ventral scale rows; the number of enlarged longitudinal ventral rows varies from the neck to the preanal scales: after the almost coadunate collar the ventral plates continue onto the upper arm and ventrally form 2 rows of pectoral scales arranged in a V-shape, there are 6 rows around the axilla, after which additional ventro-

lateral scales gradually enlarge to form up to 10 longitudinal ventral scale rows at midbody plus 1 to 2 additional rows of lateral scales smaller than the other ventrals yet larger than the dorsals adjacent to the ventrals. There are 56 [50] totally smooth, granular, oval dorsal scale rows (including the enlarged lateral scales) at the 14th transverse ventral scale row (consisting of 10 ventral plates); 4 supralabials anterior to the keeled subocular, which is much narrower beneath than above; 3 nasal scales surround the nostril which does not touch rostral or labials, lower nasal scale rhombic, pointing downwards and embedded between the anterior-most labial scale and the rostral without reaching the mouth opening; posterior nasal also in touch with the first supralabial and almost as large as the interior one; interior nasals meet in a suture; interior nasals feebly swollen; a large and narrow tympanic shield; no auricular denticulation; lower eyelid scaly and opaque. Upper head shields flat and smooth; no occipital scale or granule; frontonasal separated from the rostral by the interior nasals; 2 prefrontals, longer than broad, forming a suture in the middle; frontal longer than broad; parietals longer than broad; interparietal smaller than frontoparietals; 2 loreals; 2 complete, large central supraoculars, followed by 2 enlarged scales plus 1/2 granules anteriorly, and 3/2 enlarged scales plus 7/5 granules posteriorly, and bordered exteriorly by one row of granules in contact with the supraciliaries; posterior supraocular borders the frontoparietal and touches the frontal at the corner; 1 anterior loreal, which is barely longer than deep and shorter than the posterior one; 1 scale between subocular and posterior loreal; 5 supraciliaries, the anterior-most longest; 7 enlarged scales in collar which is distinct and free only on the sides and almost coadunate; 5 chin shields, 2 of which are fully in contact and the third up to the half; 18 scales under the 4th toe; 3 rows of scales around 4th finger and 4th toe (one dorsal, one palmar/plantar, one ulnar/fibular (outer lateral) row), the outer lateral row is serrated and consists of scales much narrower than the other two rows but does not form a distinct fringe; 16 femoral pores (which is the lowest number recorded for *A. guineensis*[4]) that meet medially.

Interestingly, a preliminary osteological investigation of ZMB 25479 revealed that the squamosal bone is in contact with the parietal bone, a condition that is supposed to be absent in *Acanthodactylus* spp.[5].

Color (in alcohol): Dorsum of dark brown tan with lighter speckles; head shields and dorsal tail lighter brown except for edges of parietals and granules posterior to the supraoculars which are of the same dark brown; dorsum and flanks with 9 cream-beige colored stripes that extend to the sacrum, becoming indistinct on the tail: 1 broad vertebral stripe flanked closely by two 2 thinner ones, 2 thin dorsolateral lines originating from the lateral edges of the parietals, and 2 (1 indistinct) thin lateral lines on each side, the upper one beginning just below the eye and the lower, broken one continues from the cream-white upper labial scales and gradually merges with the white venter; dorsal surfaces of limbs covered with creamish-white spots; ventral side of body and tail white. Sternfeld[3] mentions traces of bluish ocelli laterally which could not be detected in the preserved specimen.

Variation among the newly examined specimens

Specimen ZMB 31046 was – similar to ZMB 25479 – also recorded from the Ouham River. This time, more precisely, it was found near Bozoum (Ouham-Pendé Prefecture) in the Central African Republic by Günther Tessmann in 1914. This specimen is subadult with a SVL of 41.5 mm. The specimen has similar head shield arrangement and scalation to ZMB 25479, with a maximum of 10 longitudinal ventral scale rows; 18 scales under the 4th toe; 3 rows of scales around the 4th toe; 3 rows of scales around the 4th finger; 2 prefrontals; 4 supralabials anterior to the subocular; 5 supraciliaries; 5 chin shield pairs (3 in full contact) and 3 nasal scales arranged like described above; but differs in having 53 smooth dorsal scale rows at midbody; 27 transverse ventral scale rows; 18/17 femoral pores that meet medially; 2 complete, large central supraoculars on each side followed by 2 enlarged scales anteriorly (plus 2 granules) and 2 enlarged scales posteriorly (plus 5 granules) and bordered by 1 row of granules in contact with the supraciliaries; 10 enlarged collar scales (collar free only on the sides and almost coadunate).

The label on the jar of another specimen, ZMB 84911, denominated it as *Heliobolus nitidus* from Dŭbul, Panpamba, collected by Gaston Thierry; accessioned 23 June 1902. However, after examining this specimen we assign it to *Acanthodactylus guineensis* due to the presence of 3 rows of scales around toes and fingers (vs. 2 rows in *H. nitidus*), maximum 10 longitudinal ventral scale rows (vs. 6 rows), occipital absent (vs. present), and the arrangement of the nasal scales with the lower nasal scale embedded between the anterior-most supralabial scale and the rostral and the posterior and lower nasal in touch with the first supralabial (vs. the lower nasal resting upon the first supralabial and the posterior nasal not in contact with the supralabials[6,7]. Gaston Thierry (1866-1904) was stationed in the German colony Togoland until the year 1899 when he was relocated to Cameroon[8]. At that time the border of Togoland also comprised parts of eastern Ghana. In the atlas of the German colonies[9] we found the town name "Dĭbul" near "Bumbuna" which we believe to be the locality of specimen ZMB 84911 and which today is situated roughly 11km east of the Ghana-Togo border in Ghana (10.45°, 0.07°).

ZMB 84911 is an adult male with a SVL of 55 mm and similar head shield arrangement and scalation to ZMB 25479 with a maximum of 10 longitudinal ventral scale rows; 28 transverse ventral scale rows; 18 scales under the 4th toe; 3 rows of scales around the 4th toe; 3 rows of scales around the 4th finger; 2 prefrontals; 4 supralabials anterior to the subocular; 5 supraciliaries; 5 chin shield pairs (3 in full contact) and 3 nasal scales arranged like described above; but differs in having 62 dorsal scale rows at midbody which are smooth in the nape but from midbody onwards become slightly keeled with increasing intensity towards the tail base; 17/NA femoral pores (right leg damaged) that are separated by one scale medially; 2 complete, large central supraoculars on each side followed by 2 enlarged scales anteriorly (plus 2/1 granules) and 1 scale posteriorly (plus 4 granules) and bordered by 1 row of granules in contact with the supraciliaries (the granule row has 1 extra granule on each side outside of the linear arrangement);10 enlarged collar scales (collar free only on the sides and almost coadunate).

Specimen JFT 4143 was collected near Kouré in Niger (13.31666°, 2.5666°). It has a SVL of 49 mm and similar head shield arrangement and scalation to ZMB 25479, with a maximum of 10 longitudinal ventral scale rows; 3 rows of scales around the 4th toe; 3 rows of scales around the 4th finger; 4 supralabials anterior to the subocular; 5 chin shield pairs (3 in full contact) and 3 nasal scales arranged like described above. It differs in having 27 transverse ventral scale rows; 49 dorsal scale rows at midbody which are less granular, almost tubercular and slightly imbricate, smooth in the nape but from midbody onwards slightly keeled with increasing intensity towards the tail base; 21 scales under the 4th toe (which is one more than the maximum recorded so far[4]); 19/NA femoral pores (right leg damaged) that are separated by one scale medially; 2 complete, large central supraoculars on each side followed by 8/9 almost similar sized scales anteriorly (only the anterior-most slightly larger on each side) and 3 scales posteriorly (plus 4/5 granules) and bordered by 1 row of granules in contact with the supraciliaries; 3 prefrontals (as opposed to 2 fide[4]); 3 nasal scales a bit more bulging like the other examined specimens but arranged as described above; a very small occipital scale; only 4/3 supraciliaries (not 5).

Specimen UWBM 5965 from the Upper West Region, Gbele Resource Reserve, northwestern Ghana (10.42018, -2.07402), was accessioned at UWBM as *Acanthodactylus guineensis*. According to the original first descriptions we conclude this specimen is in fact *Heliobolus nitidus* based on the existence of only 2 rows of scales around toes and fingers, 6 longitudinal ventral scale rows, occipital present, lower nasal resting upon the first supralabial and the posterior nasal not in contact with the supralabials.

Supplementary Table S1. Mitogenomes used in this study. Provided are ID numbers used in the phylogenetic trees (ID), GenBank accession numbers (GenBank), species names (Species), clade name (Clade), genome length in base pairs (bp) and AT content (in %). Newly assembled mitogenomes are highlighted in bold.

ID	GenBank	Species	Clade	Length (bp)	AT (%)
**ZFMK59511**	**MW496123**	***Acanthodactylus guineensis***	**Eremiadini**	**16927**	**60.8**
**SB642**	**MW496124**	***Acanthodactylus schmidti***	**Eremiadini**	**17001**	**57**
**I14063**	**MW496112**	***Acanthodactylus boskianus***	**Eremiadini**	**17143**	**58**
**I14064**	**MW496113**	***Acanthodactylus erythrurus***	**Eremiadini**	**16827**	**60.9**
**I14070**	**MW496114**	***Mesalina oliveri***	**Eremiadini**	**16899**	**60**
**I14071**	**MW496115**	***Acanthodactylus aureus***	**Eremiadini**	**17021**	**59.7**
**I14083**	**MW496116**	***Acanthodactylus erythrurus***	**Eremiadini**	**16831**	**61.4**
**I14089**	**MW496118**	***Australolacerta australis***	**Eremiadini**	**17019**	**62**
**I14091**	**MW496119**	***Pedioplanis laticeps***	**Eremiadini**	**17046**	**59.8**
**I14092**	**MW496120**	***Meroles squamulosus***	**Eremiadini**	**16860**	**59.4**
**I18040**	**MW496121**	***Acanthodactylus aureus***	**Eremiadini**	**15756**	**59.1**
KJ664798	KJ664798	*Eremias multiocellata*	Eremiadini	18996	59.2
KM359148	KM359148	*Eremias velox*	Eremiadini	18033	58
KP981388	KP981388	*Eremias vermiculata*	Eremiadini	19796	60
KP981389	KP981389	*Eremias vermiculata*	Eremiadini	19494	60.2
MK261078	MK261078	*Eremias vermiculata*	Eremiadini	17972	59.4
NC_011764	NC_011764	*Eremias brenchleyi*	Eremiadini	19542	58.4
NC_016755	NC_016755	*Eremias argus*	Eremiadini	18521	58.4
NC_025304	NC_025304	*Eremias multiocellata*	Eremiadini	19385	59.5
NC_025320	NC_025320	*Eremias vermiculata*	Eremiadini	19914	59.8
NC_025929	NC_025929	*Eremias przewalskii*	Eremiadini	18225	58.2
NC_029878	NC_029878	*Eremias stummeri*	Eremiadini	19602	59.6
**I14058**	**MW496111**	***Gallotia atlantica***	**Gallotinae**	**15552**	**57.6**
**I14086**	**MW496117**	***Psammodromus algirus***	**Gallotinae**	**17118**	**60.7**
AB080237	AB080237	*Takydromus tachydromoides*	Lacertini	18245	60.3
CM020436	CM020436	*Lacerta agilis*	Lacertini	19093	61.7
**I19981**	**MW496122**	***Algyroides nigropunctatus***	**Lacertini**	**15844**	**60**
JX290083	JX290083	*Takydromus sylvaticus*	Lacertini	17838	60.3
LC101816	LC101816	*Takydromus tachydromoides*	Lacertini	17923	60.3
LR694166	LR694166	*Lacerta bilineata*	Lacertini	17147	59.6
MN122865	MN122865	*Zootoca vivipara*	Lacertini	17051	63.2
NC_008328	NC_008328	*Lacerta viridis viridis*	Lacertini	17156	59.8
NC_011606	NC_011606	*Phoenicolacerta kulzeri*	Lacertini	17199	60.5
NC_011607	NC_011607	*Podarcis muralis*	Lacertini	17311	61.4
NC_011609	NC_011609	*Podarcis siculus*	Lacertini	17297	60.8
NC_018777	NC_018777	*Takydromus wolteri*	Lacertini	18236	62
NC_021766	NC_021766	*Lacerta agilis*	Lacertini	17090	60.3
NC_022703	NC_022703	*Takydromus sexlineatus*	Lacertini	18943	62.3
NC_026867	NC_026867	*Zootoca vivipara*	Lacertini	17046	63.3
NC_028440	NC_028440	*Lacerta bilineata*	Lacertini	17086	59.7

NC_030209	NC_030209	*Takydromus amurensis*	Lacertini	17333	60
NC_045934	NC_045934	*Darevskia valentini*	Lacertini	17393	59.9
NC_046006	NC_046006	*Darevskia armeniaca*	Lacertini	17521	60.4
NC_046007	NC_046007	*Darevskia dahli*	Lacertini	17528	60.5
NC_046008	NC_046008	*Darevskia mixta*	Lacertini	17532	60.5
NC_046009	NC_046009	*Darevskia parvula*	Lacertini	17510	59.4
NC_046010	NC_046010	*Darevskia portschinskii*	Lacertini	17529	59.6
NC_046011	NC_046011	*Darevskia rudis*	Lacertini	17534	59.8
NC_046012	NC_046012	*Darevskia saxicola*	Lacertini	17524	60.2
NC_012433	NC_012433	*Blanus cinereus*	Outgroup	16969	55.6

Supplementary Table S2. Museum specimens (with accession number) and literature records (source) with coordinates in latitude (lat), longitude (lon) in decimal degrees (dd) and locality information used for reconstruction of the distribution range of *A. guineensis*. Localities used for the species distribution models and climate envelopes are highlighted in bold. * holotype *E. guineensis*; ‡ holotype *E. mandjarum*; # lectotype *E. benuensis*; ## paralectotypes *E. benuensis*.

AccessionNo/source	lat (dd)	lon (dd)	locality	country
**BM 1946.8.6.31 ***	**4.3°**	**6.25°**	**Brass mouth of the river Niger**	**Nigeria**
**ZMB 25479 ‡**	**6.3°**	**16.36°**	**Ouham**	**Central African Republic**
**MHNG 1055.62 #**	**7.9°**	**13.5833°**	**Ngayouyanga**	**Cameroon**
**MHNC 91.1008 ##**	**4.0833°**	**14.5°**	**Bangué**	**Cameroon**
MHNC 91.1007 ##	4.0833°	14.5°	Bangué	Cameroon
MHNC 91.1006 ##	4.0833°	14.5°	Bangué	Cameroon
MHNC 91.1005 ##	4.0833°	14.5°	Bangué	Cameroon
ZMB 31046	6.3°	16.36°	Ouham, Bozoum	Central African Republic
**MNHN 1996.8200**	**8.8833°**	**22.8°**	**Ouanda-Djallé**	**Central African Republic**
**Chirio 2009 (pers. comm.)**	**11.099°**	**1.756°**	**Pendjari NP, forêt de Boundjagou**	**Benin**
**ZFMK 38720**	**11.17°**	**-4.2833°**	**Fada N'Gourma**	**Burkina Faso**
ZFMK 39028	11.17°	-4.2833°	Fada N'Gourma	Burkina Faso
**ZFMK 59511**	**12.05°**	**-0.35°**	**Daroha, near Bobo Dioulasso**	**Burkina Faso**
**Trape et al. 2012**	**12.5°**	**-2.5°**	**-**	**Burkina Faso**
**Trape et al. 2012**	**7.5°**	**13.5°**	**-**	**Cameroon**
MNHN 2005.1355	7.9°	13.5833°	Ngaouyanga	Cameroon
MNHN 2005.0645	7.9°	13.5833°	Ngaouyanga	Cameroon

MNHN 2005.0644	7.9°	13.5833°	Ngaouyanga	Cameroon
**MNHN 2005.0657**	**8.5°**	**14.5°**	**Reserve de Bouban-Djija (Bouba Ndjida National Park)**	**Cameroon**
MNHN 2005.0656	8.5°	14.5°	Reserve de Bouban-Djija (Bouba Ndjida National Park)	Cameroon
MNHN 2005.0655	8.5°	14.5°	Reserve de Bouban-Djija (Bouba Ndjida National Park)	Cameroon
MNHN 2005.0642	8.5°	14.5°	Reserve de Bouban-Djija (Bouba Ndjida National Park)	Cameroon
**MNHN 2005.0647**	**10.28°**	**15.24°**	**Yagoua**	**Cameroon**
MNHN 2005.0646	10.28°	15.24°	Yagoua	Cameroon
MNHN 1999.8403	10.28°	15.24°	Yagoua	Cameroon
MNHN 1999.8402	10.28°	15.24°	Yagoua	Cameroon
**Chirio & LeBreton 2007**	**10.5°**	**13.63°**	**Mandara Mountains**	**Cameroon**
**MNHN 2005.0658**	**10.64°**	**13.78°**	**Gamba area (prob. south of Moloko)**	**Cameroon**
MNHN 2005.0643	10.64°	13.78°	Gamba area (prob. south of Moloko)	Cameroon
**MNHN 1999.8401**	**10.91°**	**13.73°**	**Fourou (or Tourou)**	**Cameroon**
**BM 1980.1010**	**10.05°**	**-2.5°**	**Wa Secondary School, Wa, Upper region**	**Ghana**
BM 1980.1009	10.05 °	-2.5 °	Wa Secondary School, Wa, Upper region	Ghana
BM 1979.612	10.05 °	-2.5 °	Wa	Ghana
BM 1979.611	10.05 °	-2.5 °	Wa	Ghana
**ZMB 84911**	**10.45 °**	**0.06667 °**	**Dĭbul, Bumbuna, Mangu Region**	**Ghana**
**BM 1966.286**	**10.6166°**	**-0.17°**	**Nakpanduri, S. of Bawku, N.E. Ghana**	**Ghana**
**ZFMK 57176**	**14.333°**	**-3.6°**	**Bandiagara**	**Mali**
**CAS 103274**	**12.4833°**	**2.4°**	**10 miles NW of Tapoa towards Tamou**	**Niger**
**JFT 4143**	**13.31666°**	**2.5666°**	**Kouré**	**Niger**
**BM (Meinig & Böhme 2002)**	**6.3°**	**5.45°**	**Kwale**	**Nigeria**
**ZMUC 45223**	**7.25°**	**9.9833°**	**Takum, Benue Province**	**Nigeria**
ZMUC 45222	7.25°	9.9833°	Takum, Benue Province	Nigeria
**ZMUC 45909**	**7.5166°**	**7.0166°**	**Lupwe, 4 ml S of Takum, Benue Province**	**Nigeria**
**ZMUC 45907**	**8.7333°**	**4.1333°**	**Igbetti**	**Nigeria**
ZMUC 45174	8.7333°	4.1333°	Igbetti Rest House	Nigeria
ZMUC 45173	8.7333°	4.1333°	Igbetti Rest House	Nigeria
ZMUC 45171	8.7333°	4.1333°	Igbetti Rest House	Nigeria
ZMUC 45170	8.7333°	4.1333°	Igbetti Rest House	Nigeria
ZMUC 45169	8.7333°	4.1333°	Igbetti Rest House	Nigeria
ZMUC 45168	8.7333°	4.1333°	Igbetti Rest House	Nigeria
ZMUC 45167	8.7333°	4.1333°	Igbetti Rest House	Nigeria
ZMUC 45166	8.7333°	4.1333°	Igbetti Rest House	Nigeria

**Meinig & Böhme 2002**	**9.35°**	**9.6667°**	**Amper**	**Nigeria**
**Meinig & Böhme 2002**	**9.35°**	**11.0333°**	**Bambur**	**Nigeria**
**ZMUC 45207**	**9.4333°**	**7.3666°**	**Idah**	**Nigeria**
ZMUC 45206	9.4333°	7.3666°	Idah	Nigeria
ZMUC 45205	9.4333°	7.3666°	Idah	Nigeria
ZMUC 45204	9.4333°	7.3666°	Idah	Nigeria
ZMUC 45203	9.4333°	7.3666°	Idah	Nigeria
ZMUC 45202	9.4333°	7.3666°	Idah	Nigeria
ZMUC 45201	9.4333°	7.3666°	Idah	Nigeria
ZMUC 45200	9.4333°	7.3666°	Idah	Nigeria
ZMUC 45199	9.4333°	7.3666°	Idah	Nigeria
ZMUC 45191	9.4333°	7.3666°	Idah	Nigeria
ZMUC 45190	9.4333°	7.3666°	Idah	Nigeria
ZMUC 45189	9.4333°	7.3666°	Idah	Nigeria
ZMUC 45179	9.4333°	7.3666°	Idah Rest House	Nigeria
ZMUC 45178	9.4333°	7.3666°	Idah Rest House	Nigeria
ZMUC 45177	9.4333°	7.3666°	Idah Rest House	Nigeria
ZMUC 45176	9.4333°	7.3666°	Idah Rest House	Nigeria
ZMUC 45175	9.4333°	7.3666°	Idah Rest House	Nigeria
**ZMUC 45196**	**9.6333°**	**8.75°**	**Riyom, Jos Plateau**	**Nigeria**
ZMUC 45195	9.6333°	8.75°	Riyom, Jos Plateau	Nigeria
ZMUC 45194	9.6333°	8.75°	Riyom, Jos Plateau	Nigeria
ZMUC 45193	9.6333°	8.75°	Riyom, Jos Plateau	Nigeria
ZMUC 45188	9.6333°	8.75°	Riyom, Jos Plateau	Nigeria
ZMUC 45187	9.6333°	8.75°	Riyom, Jos Plateau	Nigeria
ZMUC 45186	9.6333°	8.75°	Riyom, Jos Plateau	Nigeria
ZMUC 45185	9.6333°	8.75°	Riyom, Jos Plateau	Nigeria
ZMUC 45184	9.6333°	8.75°	Riyom, Jos Plateau	Nigeria
**BM 1962.575**	**9.78333°**	**8.2666°**	**Zonkwa, S. Zaria**	**Nigeria**
BM 1961.952	9.78333°	8.2666°	Zonkwa, S. Zaria, N. Nigeria	Nigeria
BM 1961.2000	9.78333°	8.2666°	Zonkwa, S. Zaria, N. Nigeria	Nigeria
BM 1961.1999	9.78333°	8.2666°	Zonkwa, S. Zaria, N. Nigeria	Nigeria
BM 1961.1998	9.78333°	8.2666°	Zonkwa, S. Zaria, N. Nigeria	Nigeria
BM 1961.1997	9.78333°	8.2666°	Zonkwa, S. Zaria, N. Nigeria	Nigeria
BM 1961.1996	9.78333°	8.2666°	Zonkwa, S. Zaria, N. Nigeria	Nigeria
BM 1961.1995	9.78333°	8.2666°	Zonkwa, S. Zaria, N. Nigeria	Nigeria
BM 1961.1994	9.78333°	8.2666°	Zonkwa, S. Zaria, N. Nigeria	Nigeria
BM 1961.1993	9.78333°	8.2666°	Zonkwa, S. Zaria, N. Nigeria	Nigeria
BM 1961.1992	9.78333°	8.2666°	Zonkwa, S. Zaria, N. Nigeria	Nigeria
BM 1961.1991	9.78333°	8.2666°	Zonkwa, S. Zaria, N. Nigeria	Nigeria
BM 1961.1990	9.78333 °	8.2666°	Zonkwa, S. Zaria, N. Nigeria	Nigeria
**BM 1962.1661**	**9.91°**	**8.88°**	**10.5 miles southwest of Jos, Plateau Province, N. Nigeria**	**Nigeria**
**Meinig & Böhme 2002**	**10.3°**	**9.833°**	**Bauchi**	**Nigeria**

**BM 1973.660**	**11.08333°**	**7.7°**	**Zaria, N.C. State**	**Nigeria**
BM 1962.1664	11.08333°	7.7°	Vet. Unit, Zonkwa, Zaria Province, N. Nigeria	Nigeria
BM 1962.1663	11.08333°	7.7°	Vet. Unit, Zonkwa, Zaria Province, N. Nigeria	Nigeria
BM 1962.1662	11.08333°	7.7°	Vet. Unit, Zonkwa, Zaria Province, N. Nigeria	Nigeria
**BM 1930.10.6.9**	**11.78°**	**9.6°**	**Kigawa River (probably Kiyawa), near Shenfuri (Sherifuri following Dunger 1967), N. Nigeria**	**Nigeria**
**BM 1962.1669**	**11.85°**	**13.15°**	**Maiduguri, Bornu Province, N. Nigeria**	**Nigeria**
BM 1962.1668	11.85°	13.15°	Maiduguri, Bornu Province, N. Nigeria	Nigeria
BM 1962.1667	11.85°	13.15°	Maiduguri, Bornu Province, N. Nigeria	Nigeria
BM 1962.1666	11.85°	13.15°	Maiduguri, Bornu Province, N. Nigeria	Nigeria
BM 1962.1665	11.85°	13.15°	Maiduguri, Bornu Province, N. Nigeria	Nigeria
**BM 1962.572**	**12°**	**8.5°**	**Kano (northern Nigeria)**	**Nigeria**

Supplementary Table S3. Best fit nucleotide substitution models and partition scheme selected by the ModelFinder algorithm in iQTree for the alignment of DNA sequences from 50 terminals, including 14,241 sites of 12S and 16S rRNAs plus the 13 protein-coding genes extracted from mitogenomic assemblies of lacertids. Columns show for each partition, the number of taxa for which the partition was available (#Seqs), the number of nucleotide positions (#Sites), the number of site patterns (#Patterns), and the percentage of invariable sites (#Const.Sites).

Model	Partition	#Seqs	#Sites	#Patterns	#Const.Sites
GTR+R5	rrnL + rrnS + atp6(codon1) + nad4L(codon1) + atp8(codon2) + nad2(codon1) + nad4(codon1) + cytb(codon1) + nad1(codon1) + nad3(codon1) + atp8(codon1) + nad6(codon1)	50	4,986	2,886	40%
TVM+R4	atp6(codon2) + nad4L(codon2) + nad2(codon2) + nad3(codon2) + nad4(codon2) + nad5(codon2) + cox1(codon2) + cox2(codon2) + cox3(codon2) + cytb(codon2) + nad1(codon2) + nad6(codon2) + cox1(codon1) + cox2(codon1) + cox3(codon1)	50	4,801	1,335	68%
GTR+R6	atp6(codon3) + cytb(codon3) + nad2(codon3) + cox1(codon3) + cox2(codon3) + nad4L(codon3) + nad3(codon3) + nad4(codon3) + nad5(codon3) + cox3(codon3) + nad1(codon3) + atp8(codon3)	50	3,660	3,590	2%
GTR+I+G4	nad5(codon1)	50	610	328	42%
TN+R3	nad6(codon3)	50	184	179	7%

Supplementary Table S4. Nucleotide substitution models and partition scheme used for Bayesian Inference, as selected by the ModelFinder algorithm in iQTree (constrained to models implemented in MrBayes) for the alignment of DNA sequences from 50 terminals, including 14,241 sites of 12S and 16S rRNAs plus the 13 protein-coding genes extracted from mitogenomic assemblies of lacertids.

Model	Partition
GTR+I+G	rrnL + rrnS + atp6(codon1) + atp8(codon2) + nad4L(codon1) + nad2(codon1) + nad4(codon1) + cytb(codon1) + nad1(codon1) + nad3(codon1) + atp8(codon1) + nad6(codon1)
GTR+I+G	atp6(codon2) + nad4L(codon2) + nad2(codon2) + nad3(codon2) + nad4(codon2) + nad5(codon2) + cox1(codon2) + cox2(codon2) + cox3(codon2) + cytb(codon2) + nad1(codon2) + nad6(codon2) + cox1(codon1) + cox2(codon1) + cox3(codon1)
GTR+I+G	atp6(codon3) + cox2(codon3) + nad1(codon3) + nad3(codon3) + nad4(codon3) + nad5(codon3) + cox1(codon3)
HKY+I+G	atp8(codon3) + nad4L(codon3)
GTR+I+G	cox3(codon3) + cytb(codon3) + nad2(codon3)
GTR+I+G	nad5(codon1)
HKY+I+G	nad6(codon3)

Supplementary Table S5. Best fit nucleotide substitution models and partition scheme selected by the ModelFinder algorithm in iQTree for the more taxa comprehensive alignment, with reduced gene coverage, including 3,054 sites of the 12S, 16S, COB and ND4 genes from 250 terminals of Lacertidae. Columns show for each partition, the number of taxa for which the partition was available (#Seqs), the number of nucleotide positions (#Sites), the number of site patterns (#Patterns), and the percentage of invariable sites (#Const.Sites).

Model	Partition	#Seqs	#Sites	#Patterns	#Const.Sites
TVM+R6	12S rRNA	204	410	287	40%
TIM2+R7	16S rRNA	190	592	425	43%
TVM+R7	COB (codon 1)	236	380	282	48%
TVM+R4	COB (codon 2)	236	380	240	61%
GTR+R6	COB (codon 3)	236	380	380	0%
TPM3u+R5	ND4 (codon 1)	126	304	232	33%
TIM3+R4	ND4 (codon 2)	126	304	186	48%
TIM3+R5	ND4 (codon 3)	126	304	297	5%

Supplementary Table S6. Locality records in latitude and longitude added from the literature (source) for taxa that were not represented in the GBIF database.

Taxon	latitude(dd)	longitude(dd)	source
Acanthodactylus ahmaddisii	31.50°	36.00°	[10]
Acanthodactylus boueti	10.23°	0.68°	[11]
Acanthodactylus harranensis	36.85°	39.00°	[12]
Acanthodactylus lacrymae	32.61°	-4.51°	[13]
“	32.18222°	-5.20532°	[13]
“	32.3763°	-5.1797°	[13]
“	32.53°	-5.19°	[13]
“	32.3°	-5.42°	[13]
“	32.1986°	-5.6292°	[13]
“	32.2182°	-5.5501°	[13]
Acanthodactylus montanus	30.69°	-7.77°	[13]
“	30.74°	-7.81°	[13]
“	30.747°	-7.6093°	[13]
“	30.68°	-7.58°	[13]
“	31.2879°	-7.3824°	[13]
Acanthodactylus orientalis	30.99°	46.33°	[14]
Acanthodactylus yemenicus	13.56°	44.03°	[15]
“	12.76°	45.01°	[15]
“	12.86°	44.98°	[15]

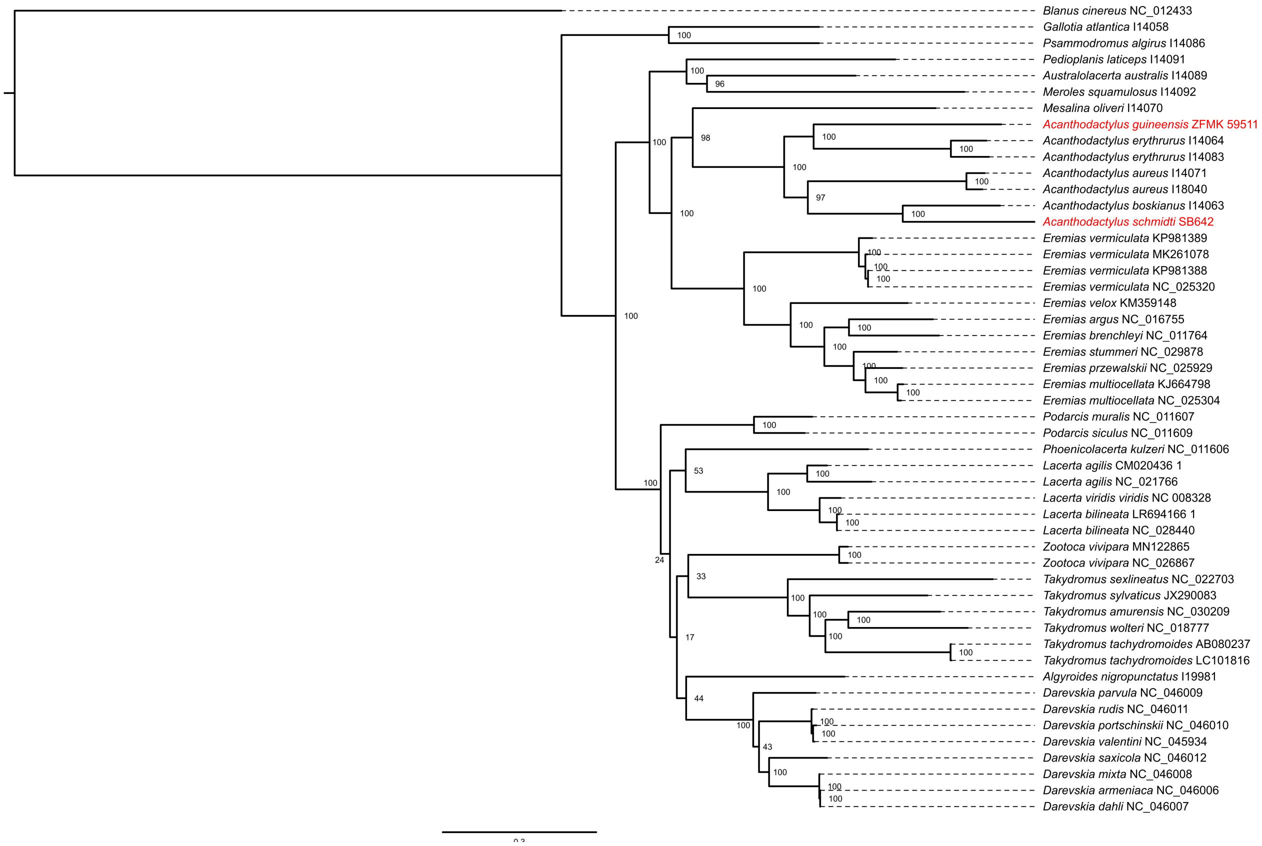

Supplementary Figure S1. Phylogenetic reconstruction of Lacertidae relationships with selected taxa representing major tribes using maximum likelihood obtained in IQTree from the high gene-coverage alignment of DNA sequences from 50 terminals, including 14,241 sites of 12S and 16S rRNAs and 13 protein-coding genes extracted from mitogenomic assemblies of lacertids. Focal taxa are highlighted in red.

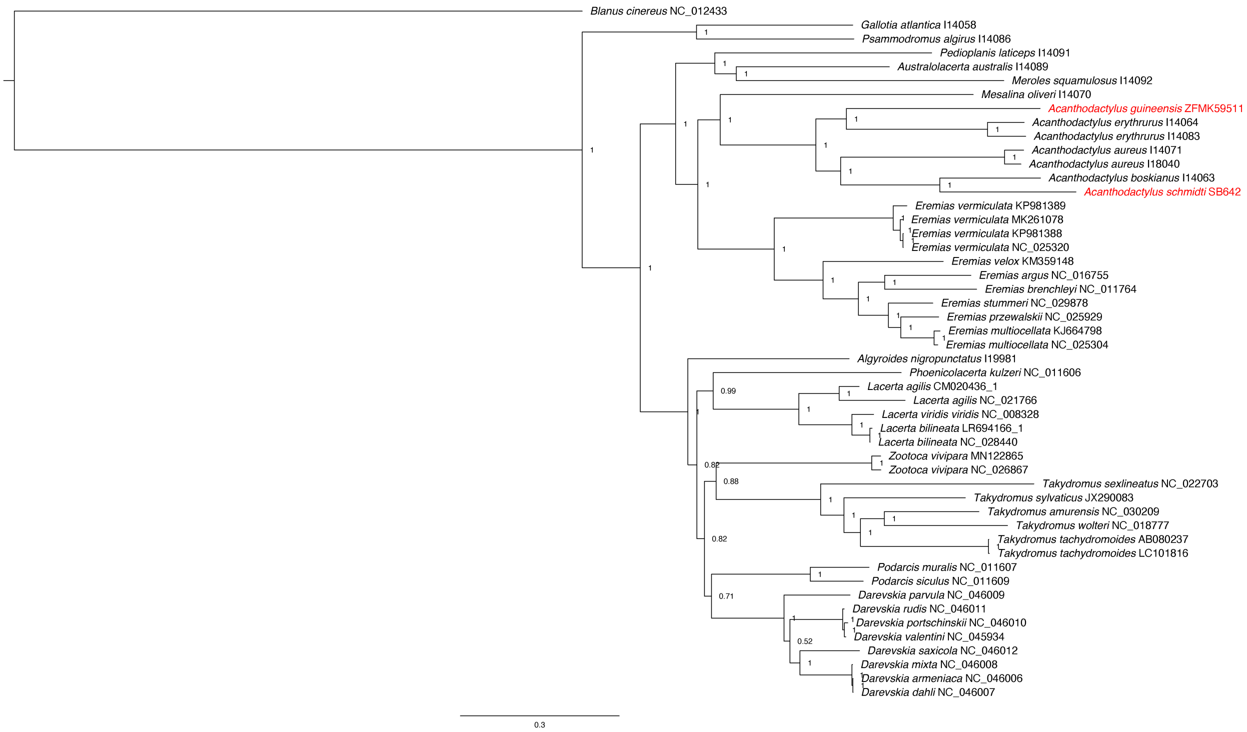

Supplementary Figure S2. Bayesian inference of the phylogenetic relationships between lacertids obtained in MrBayes from the high gene-coverage alignment of DNA sequences from 50 terminals, including 14,241 sites of 12S and 16S rRNAs and 13 protein-coding genes extracted from mitogenomic assemblies of lacertids. The tree represents the consensus of all post-burning trees with posterior probabilities indicated next to each node. Focal taxa are highlighted in red.

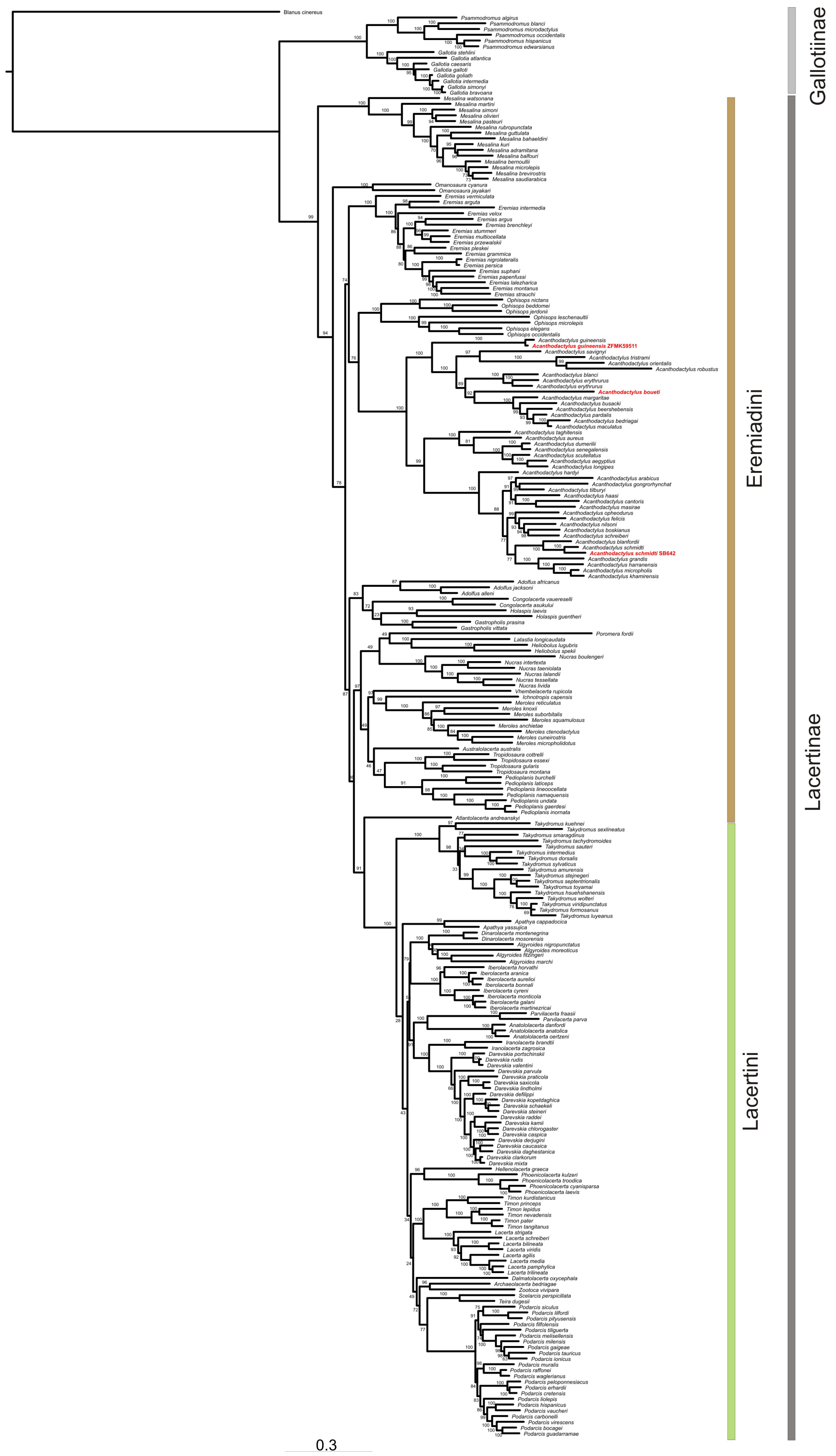

Supplementary Figure S3. Phylogenetic reconstruction obtained in IQTree from the more taxa comprehensive lacertid alignment, with reduced gene coverage, including 3,054 sites of the 12S, 16S, COB and ND4 genes from 250 terminals. Focal taxa are highlighted in red.

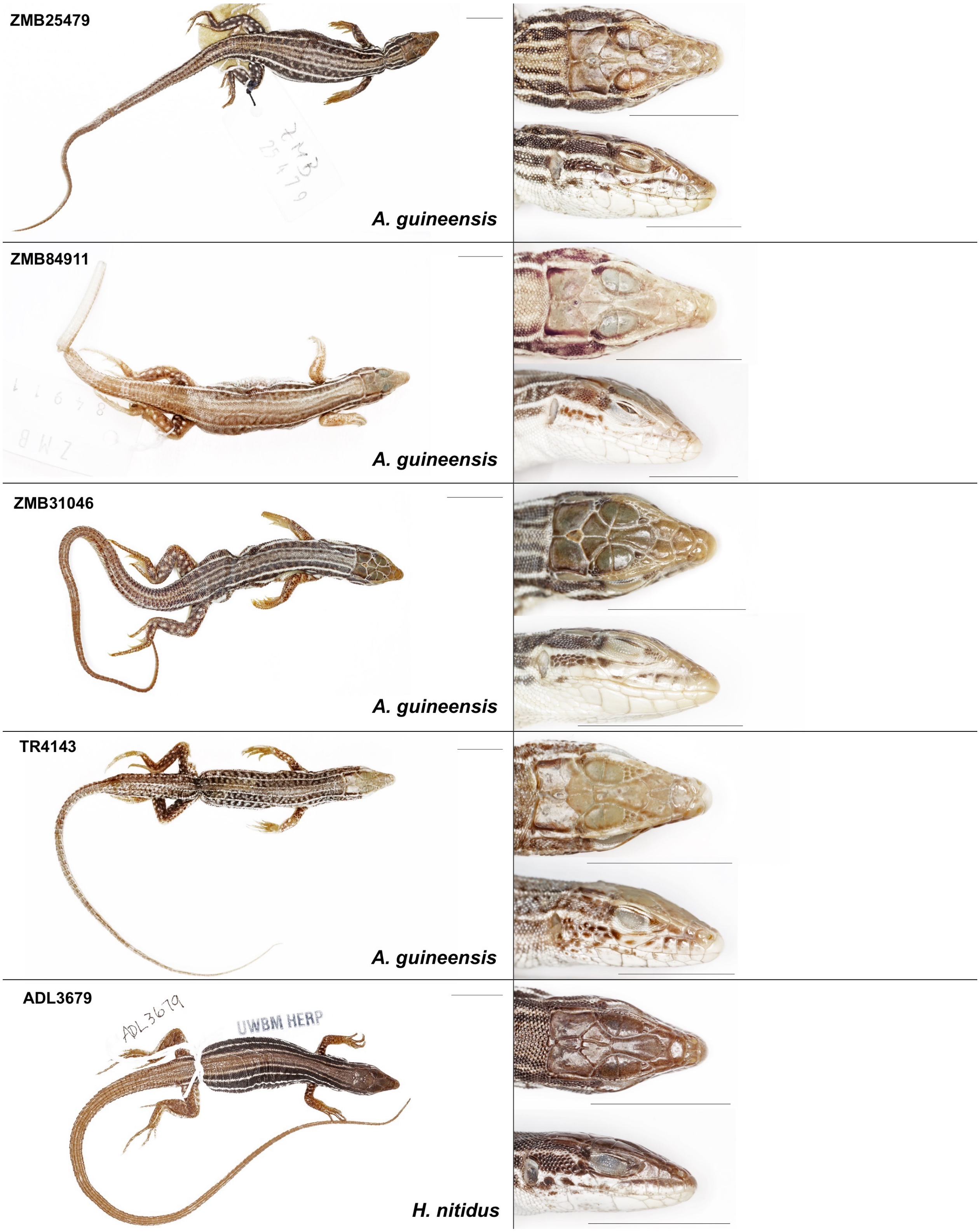

Supplementary Figure S4. Photographs of specimens examined in detail over the course of this study. The black bars indicate 1 cm. Photographs taken by S. Kirchhof.

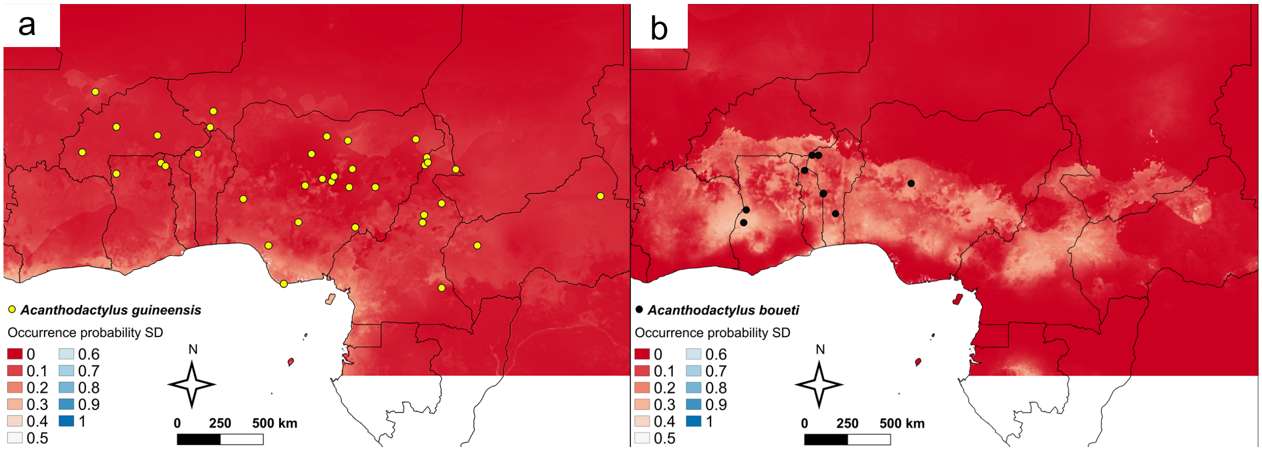

Supplementary Figure S5 a-b. Standard deviations of occurrence probabilities (higher with colder colors) calculated for the Maxent models of *A. guineensis* (a;○) and *A. boueti* (b;●) based on environmental parameters. Areas in white were not modeled. The figure was created using QGIS 3.14.16 (https://www.qgis.org).

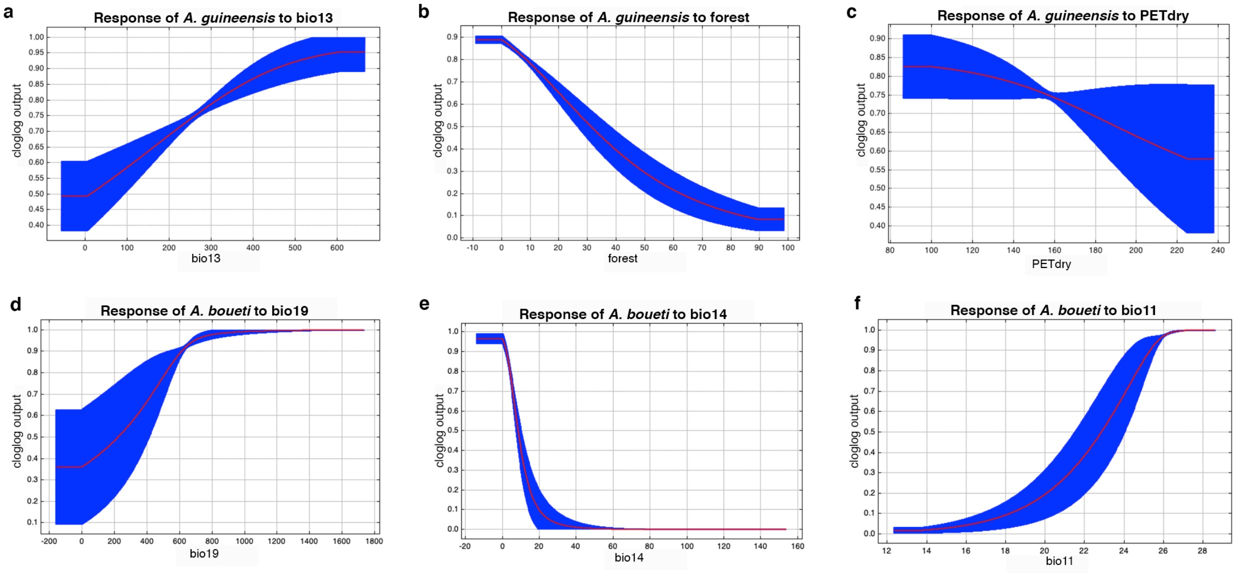

Supplementary Figure S6 a-f. Maxent response plots for *A. guineensis* (a-c) and *A. boueti* (d-f) to the respective most contributing environmental parameters (bio13 = Precipitation of wettest week (mm); forest = forest cover per grid cell (%); PETdry = potential evaporation of the driest quarter; bio19 = Precipitation of coldest quarter (mm); bio14 = Precipitation of driest week (mm); bio11 = Mean temperature of coldest quarter (°C)).

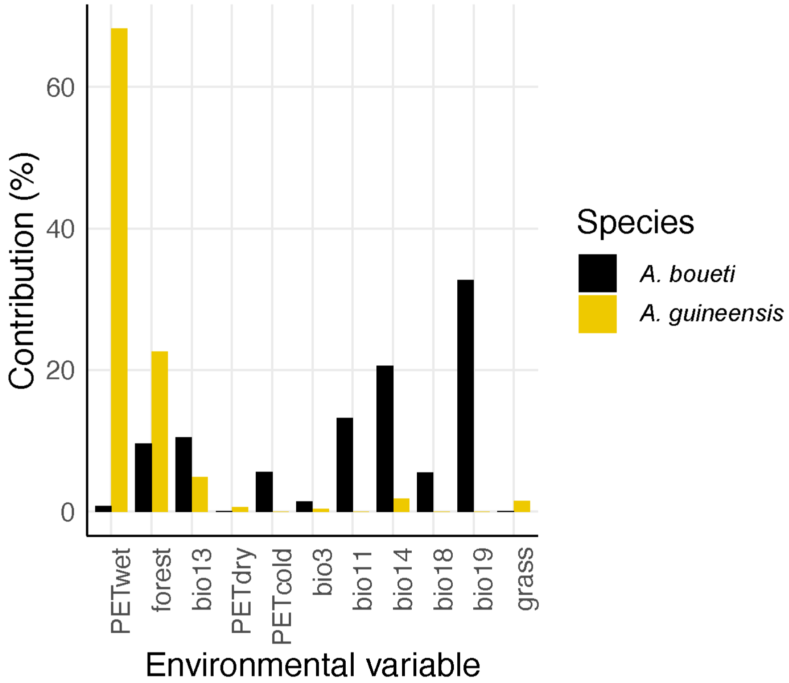

Supplementary Figure S7. Contributions in percent of the environmental variables used in the respective Maxent models for *A. boueti* and *A. guineensis.* (PETwet = potential evapotranspiration during the wettest quarter; forest = forest cover per grid cell (%); bio13 = Precipitation of wettest week (mm); PETdry = potential evapotranspiration during the driest quarter; PETcold = potential evapotranspiration during the coldest quarter; bio3 = Isothermality; bio11 = Mean temperature of coldest quarter (°C); bio14 = Precipitation of driest week (mm); bio18 = Precipitation of warmest quarter (mm); bio19 = Precipitation of coldest quarter (mm); grass = grassland, scrub and woodland (grass) per grid cell).

References

1 Chirio, L. & LeBreton, M. *Atlas des reptiles du Cameroun*. Vol. 67 (IRD Editions, 2007).

2 LeBreton, M. *A working checklist of the herpetofauna of Cameroon. With localities for species occurring in Southern Cameroon and a list of herpetofauna for the Dja Faunal Reserve.* IUCN, Amsterdam, Netherlands (1999).

3 Sternfeld, R. Zwei neue Eidechsen aus Neukamerun. *Sitzungsber. Ges. Naturf. Freunde Berlin*, 173–174 (1916).

4 Meinig, H. & Böhme, W. A note on *Acanthodactylus guineensis* (Boulenger, 1887) (Sauria: Lacertidae). *Rev. Suisse Zool.* **109,** 551–558 (2002).

5 Arnold, E. N. Towards a phylogeny and biogeography of the Lacertidae: relationships within an Old-World family of lizards derived from morphology. *Bull. Br. Museum Nat. Hist. Zool.* **55,** 209–257 (1989).

6 Günther, A. Description of three new species of Eremias. *J. Nat. Hist.* **9,** 381–382 (1872).

7 Boulenger, G. V. Descriptions of new reptiles and batrachians in the British Museum (Natural History).—Part III. *J. Nat. Hist.* **20,** 50–53 (1887).

8 Zurstrassen, B. *Die Steuerung und Kontrolle der kolonialen Verwaltung und ihrer Beamten am Beispiel des" Schutzgebietes" Togo (1884-1914)* PhD thesis. (Bundeswehr München, 2005).

9 Sprigade, P. & Moisel, M. *Grosser Deutscher Kolonialatlas*. (Kolonialabtheilung des Auswärtigen Amts, 2002).

10 Meiri, S. *et al.* Extinct, obscure or imaginary: The lizard species with the smallest ranges. *Divers. Distrib.* **24,** 262–273. https://doi.org/10.1111/ddi.12678 (2018).

11 Segniagbeto, G. H. *et al.* Checklist of the lizards of Togo (West Africa), with comments on systematics, distribution, ecology, and conservation. *Zoosystema* **37,** 381–402. https://doi.org/10.5252/z2015n2a7 (2015).

12 Baran, İ. *et al. Acanthodactylus harranensis*, a new species of lizard from southeastern Turkey (Reptilia: Sauria: Lacertidae). *Boll. Mus. Reg. Sci. Nat. Torino* **23,** 323–341 (2005).

13 Miralles, A. *et al.* Morphology and multilocus phylogeny of the Spiny-footed Lizard (*Acanthodactylus erythrurus*) complex reveal two new mountain species from the Moroccan Atlas. *Zootaxa* **4747.** https://doi.org/10.11646/zootaxa.4747.2.4 (2020).

14 Mohammed, R. G., Rhadi, F. A., Rastegar-Pouyani, N., Rastegar-Pouyani, E. & Yousefkhani, S. H. Zoogeography of lizards fauna from central and southern Iraq with a checklist of Iraqi lizard's fauna. *Russ. J. Herpetol* **24,** 193–201 (2017).

15 Salvador, A. *A revision of the lizards of the genus* Acanthodactylus *(Sauria: Lacertidae)*. (Zoologisches Forschungsinstitut und Museum Alexander Koenig Bonn, 1982).
